# Humanized and Charge‐Optimized CSPG4‐Specific CAR‐T Cells show Enhanced Efficacy against Head and Neck Squamous Cell Carcinoma

**DOI:** 10.1002/advs.202519746

**Published:** 2026-02-16

**Authors:** Xiang Xu, Shizhen Qiu, Zhitong Wang, Dan Li, Min Chen, Yinying Chu, Guangfei Li, Yi Fang, Changjiang Li, Fang Shi, Peijie He, Haitao Wu, Haopeng Wang, Jian Chen

**Affiliations:** ^1^ ENT Institute and Department of Otorhinolaryngology, Eye & ENT Hospital Fudan University Shanghai China; ^2^ Shanghai Key Laboratory of Gene Editing and Cell Therapy for Rare Diseases Fudan University Shanghai China; ^3^ Department of Hematology, Shanghai General Hospital Shanghai Jiaotong University School of Medicine Shanghai China; ^4^ School of Life Science and Technology ShanghaiTech University Shanghai China; ^5^ Changping Laboratory Beijing China

**Keywords:** CAR‐T, CSPG4, HNSCC, humanization, tonic signaling

## Abstract

Major challenges of developing CAR‑T cell therapy for head and neck squamous cell carcinoma (HNSCC) include identifying a robust tumor antigen and a suitable CAR design. Here, we validate chondroitin sulfate proteoglycan 4 (CSPG4) as a highly expressed, prognostic antigen in HPV‑negative HNSCC that drives tumor proliferation. By grafting the murine single‐chain variable fragment (scFv) 763.74 complementarity determining regions (CDRs) onto a human antibody framework engineered to minimize surface positive‑charge patches and immunogenic epitopes, we generated humanized CSPG4 (CSPG4^Hu^) CAR‑T cells with reduced tonic signaling and alleviated exhaustion. Transcriptomic and metabolic profiling reveal that this biophysical refinement reprograms CAR‐T cells away from a glycolytic, terminal exhaustion state toward a PI3K/Akt‐driven stem‐like state. Consequently, CSPG4^Hu^ CAR‐T cells demonstrate superior persistence and potent antitumor efficacy across systemic xenograft and patient‐derived xenograft models. Our study establishes a rational engineering framework that links biophysical CAR design to transcriptomic and metabolic rejuvenation, offering a promising therapeutic candidate for advanced HNSCC.

## Graphical abstract

CSPG4 is identified as a high‐value, stemness‐associated target in HPV‐negative HNSCC. By implementing rational biophysical engineering, a humanized and charge‐optimized CAR is developed to overcome tonic signaling‐induced exhaustion. This strategy induces a profound transcriptomic shift toward a rejuvenated, stem‐like memory state, significantly enhancing metabolic fitness and antitumor durability in multiple HNSCC models.

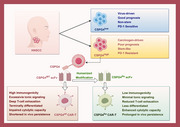

## 1 Introduction

Head and neck squamous cell carcinoma (HNSCC) is an aggressive epithelial malignancy with an estimated annual global incidence exceeding 600 000 cases and high morbidity and mortality, particularly among HPV‐negative patients [1, 2]. Despite advances in surgery, radiotherapy, and chemotherapy, the 5‐year overall survival for advanced‐stage HNSCC remains below 50%, and options for recurrent or metastatic disease are limited [3]. Immune checkpoint blockade with anti‐PD‐1 antibodies has shown encouraging clinical responses. However, its efficacy is curtailed by the profoundly immunosuppressive tumor microenvironment and pronounced intra‐ and intertumoral heterogeneity [3, 4]. These challenges underscore the urgent need for novel, targeted immunotherapies capable of overcoming inhibitory signals, achieving robust tumor specificity, and maintaining durable antitumor activity in HNSCC.

Chimeric antigen receptor (CAR) T cell therapy has revolutionized the treatment of hematologic malignancies [5, 6], yet its application in solid tumors remains challenging due to tumor heterogeneity, limited T cell persistence, antigen escape, and an immunosuppressive microenvironment [7, 8]. Progress in CAR‐T therapy for HNSCC has been particularly slow, largely because of the absence of truly tumor‐specific antigens. Chondroitin sulfate proteoglycan 4 (CSPG4) is a cell‐surface proteoglycan overexpressed in a wide range of solid tumors, including HNSCC, but with minimal expression in normal tissues, making it an attractive immunotherapeutic target [9]. However, the value of CSPG4 as a CAR‐T target in HNSCC has not been rigorously evaluated. Furthermore, traditional CAR‐T constructs derived from murine single‐chain variable fragments (scFvs), such as the widely used 763.74 clone, often suffer from high immunogenicity and deleterious biophysical properties. Specifically, suboptimal surface charge distributions can trigger antigen‐independent CAR clustering and chronic tonic signaling, ultimately driving premature functional exhaustion and severely limiting long‐term CAR‐T persistence [10, 11, 12].

To address these challenges, we first validated CSPG4 as a clinically relevant HNSCC antigen by demonstrating its overexpression in HPV‐negative tumors, its association with poor patient survival, and its role in driving tumor proliferation and progression. We then employed a rational biophysical engineering strategy to humanize and charge‐optimize the murine scFv. Our findings demonstrate that this optimization does not merely reduce immunogenicity but induces a profound transcriptomic and metabolic reprogramming of the CAR‐T cells. By attenuating tonic signaling, the humanized CSPG4.CAR‐T cells maintained a stem‐like memory signature and superior metabolic fitness, leading to enhanced persistence and superior tumor regression in both cell line‐derived and patient‐derived xenograft (PDX) models. Together, our findings establish CSPG4 as a promising therapeutic target and outline a next‐generation CAR‐T engineering strategy to advance HNSCC immunotherapy.

## 2 Results

### 2.1 CSPG4 is Enriched in HPV‑Negative HNSCC and Correlates with Poor Prognosis

**FIGURE 1:**
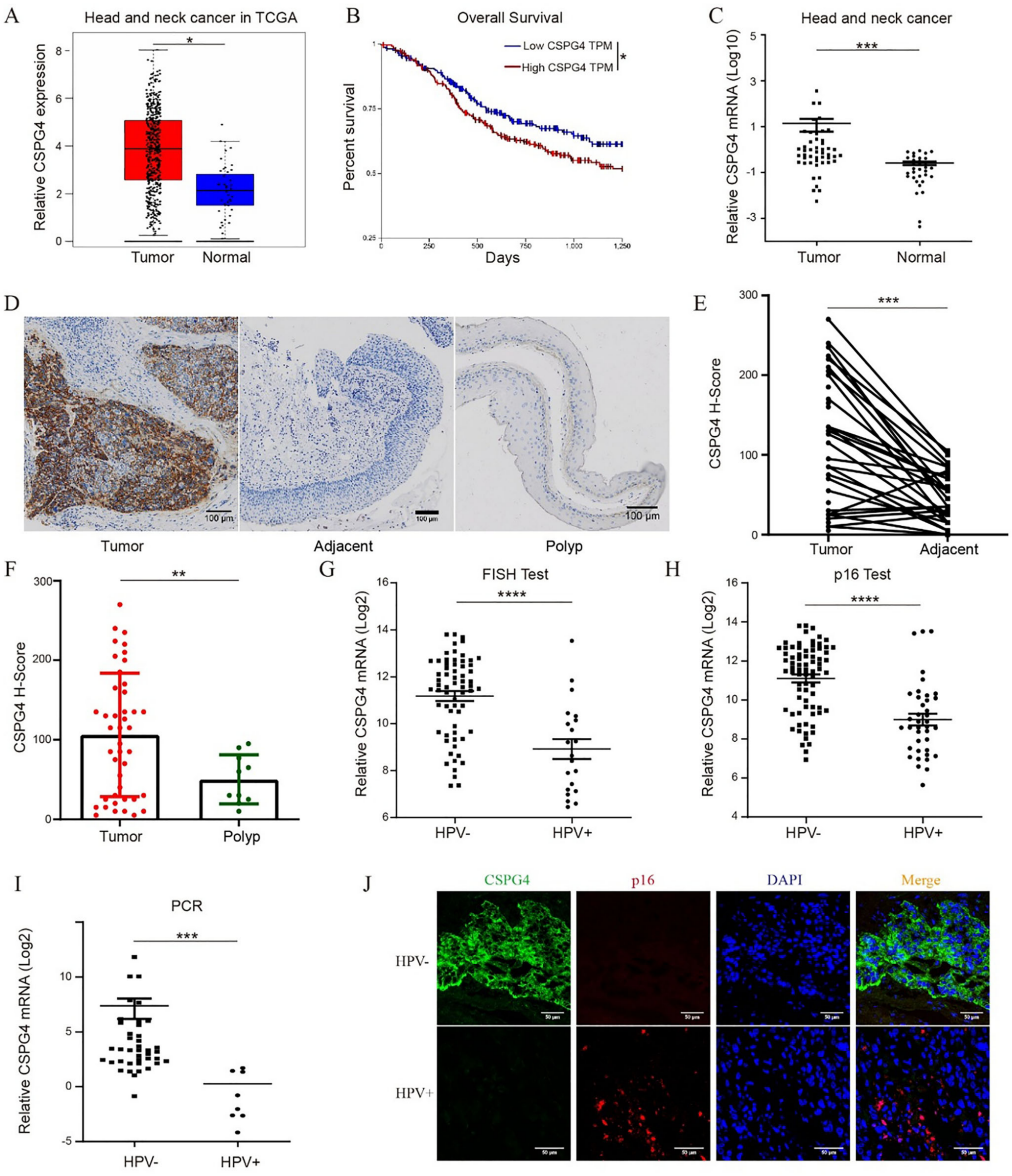
CSPG4 is overexpressed in HNSCC, correlates with poor prognosis, and is enriched in HPV‐negative tumors. (A) Comparison of CSPG4 mRNA levels between HNSCC tumors and normal head and neck tissues in TCGA. (B) Kaplan–Meier analysis of overall survival for TCGA HNSCC patients stratified by high vs. low CSPG4 transcript levels. (C) qPCR quantification of CSPG4 mRNA in paired surgical HNSCC specimens and adjacent non‐tumor tissues from our clinical cohort. (D) Representative immunohistochemical staining for CSPG4 protein in HNSCC, adjacent normal mucosa, and benign vocal‐cord polyp tissues (Scale bar = 100 µm). (E) H‐score comparison of CSPG4 IHC between HNSCC tumors and matched adjacent tissues. (F) H‐score comparison of CSPG4 IHC between HNSCC tumors and benign vocal‐cord polyp tissues. (G) Stratification of CSPG4 mRNA levels in TCGA HNSCC samples categorized as HPV‐positive or HPV‐negative by FISH. (H) CSPG4 mRNA levels in TCGA HNSCC samples categorized as HPV‐positive or HPV‐negative by p16 IHC. (I) qPCR analysis of CSPG4 mRNA in surgical HNSCC specimens classified by PCR‐based HPV status. (J) Representative immunofluorescence for CSPG4 (green), p16 (red), and DAPI (blue) in HPV‐positive and HPV‐negative HNSCC sections (Scale bar = 50 µm). Error bars represent Mean ± SEM. Statistical significance was determined by Student's *t*‐test or log‐rank test (^*^ *p* < 0.05, ^**^ *p* < 0.01, ^***^ *p* < 0.001, ^****^ *p* < 0.0001).

We first queried CSPG4 expression in The Cancer Genome Atlas (TCGA) and found that CSPG4 mRNA levels were significantly higher in HNSCC tumors than in normal head and neck tissues (Figure 1A). Kaplan–Meier survival analysis demonstrated that patients with high CSPG4 expression exhibited markedly worse overall survival (Figure 1B). These findings were validated in our cohort by qPCR, which showed elevated CSPG4 transcripts in surgical tumor specimens vs. adjacent controls (Figure 1C), and immunohistochemistry revealed intense CSPG4 protein staining and higher Histochemistry scores (H‐scores) in HNSCC tissues compared with both adjacent normal mucosa and benign polyp tissues (Figure 1D–F).

Head and neck tumors are classified as HPV‐positive or HPV‐negative [13]. Since HPV‐negative cases are more prevalent and typically carry a poorer prognosis [14], we next assessed CSPG4 expression across these subtypes. Stratification of TCGA cases by HPV status using FISH demonstrated that HPV‐negative tumors had inferior survival (Figure S1A) and significantly higher CSPG4 mRNA levels than HPV‐positive tumors (Figure 1G), a pattern recapitulated when HPV status was defined by p16 IHC (Figure 1H; Figure S1B) and confirmed by PCR in our specimens (Figure 1I). Co‐immunofluorescence further visualized that CSPG4 protein was predominantly expressed in HPV‐negative tumor regions lacking p16 signal (Figure 1J).

To evaluate CSPG4 expression in vitro, we analyzed a panel of ten HNSCC cell lines, FaDu, Cal‑27, HN6, HN‑8, HEp‑2, CNE‑1, CNE‑2, HNE‑2, 5‑8F, and 6‑10B, alongside the normal nasopharyngeal epithelial line NP69 as a control. qPCR revealed minimal CSPG4 transcripts in NP69, whereas the majority of HNSCC lines showed significantly elevated mRNA levels (Figure S1C). Western blotting confirmed negligible CSPG4 protein expression in NP69, with pronounced overexpression in HN‑8, HEp‑2, CNE‑2, HNE‑2, 5‑8F, and 6‑10B (Figure S1D). Flow cytometry further substantiated these findings, showing no detectable surface expression on NP69 but strong membrane staining on multiple tumor lines (Figure S1E). Quantitative analysis confirmed significantly higher CSPG4 levels in these tumor lines compared with NP69 (Figure S1F).

Cancer stem cells (CSCs) are implicated in resistance to radiotherapy and chemotherapy [15], as well as in recurrence and metastasis of solid tumors [16], and prior studies have shown that CD44‐positive HNSCC cells exhibit CSC‐like properties [17]. We therefore examined the relationship between CSPG4 and the stem‐cell marker CD44. Correlation analysis of TCGA data revealed a significant positive association between the expression of these two markers (Figure S1G). Consistent with this, immunofluorescence on patient tissues and flow cytometry profiling of in cell lines then confirmed widespread co‐expression of CSPG4 and CD44 (Figure S1H,I), suggesting that CSPG4 may serve as a hallmark of the CSC‐like population in HNSCC.

### 2.2 CSPG4 Promotes Proliferation, Stemness, and Tumorigenesis in HNSCC Cells

**FIGURE 2:**
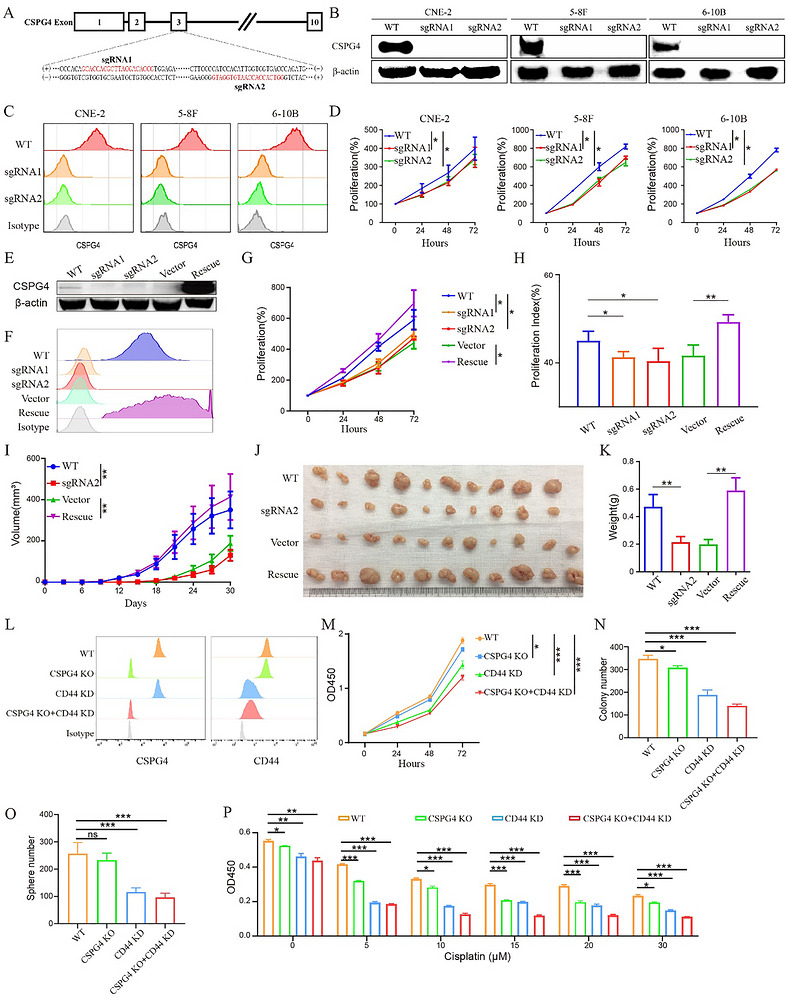
CSPG4 drives HNSCC proliferation and tumorigenesis and functionally cooperates with CD44 to maintain stemness. (A) Schematic of the CRISPR/Cas9 strategy targeting exon 3 of the CSPG4 gene. (B) Western blot confirming CSPG4 protein knockout in three HNSCC cell lines. (C) Flow cytometric histograms demonstrating loss of surface CSPG4 expression in CSPG4‐KO cells. (D) In vitro proliferation kinetics of CSPG4‐KO vs. wild‐type HNSCC cell lines. (E) Western blot validating stable CSPG4 reconstitution (Rescue) in CSPG4‐KO CNE‐2 cells. (F) Flow cytometric verification of surface CSPG4 restoration in Rescue cells. (G) In vitro proliferation assay showing the recovery of growth rates in Rescue cells. (H) Cell‐cycle distribution analysis and calculated proliferation index of Rescue cells. (I) Measurement of subcutaneous tumor volumes in NSG mice. (J) Representative photographs of excised tumors at the study endpoint. (K) Quantitative comparison of terminal tumor weights among the indicated groups. (L) Flow cytometric histograms showing surface expression of CSPG4 and CD44 in isogenic cell lines. (M) Quantitative assessment of cell proliferation in isogenic CNE‐2 cell models. (N) Colony‐formation capacity of the indicated isogenic HNSCC cell lines. (O) Statistical quantification of oncosphere formation efficiency in the indicated isogenic lines. (P) Dose‐response curves illustrating cisplatin sensitivity across isogenic cell models. Error bars represent Mean ± SEM. Statistical significance was determined by one‐way or two‐way ANOVA (^*^ *p* < 0.05, ^**^ *p* < 0.01, ^***^ *p* < 0.001).

To interrogate the functional role of CPSG4 in HNSCC, we first employed a CRISPR/Cas9‐mediated gene knockout (KO) strategy targeting exon 3 of the CSPG4 gene (Figure 2A). We successfully generated CSPG4‐KO lines in three independent HNSCC cell models. Western blot confirmed the complete ablation of CSPG4 protein expression (Figure 2B), and flow cytometry verified the concomitant abrogation of surface expression (Figure 2C). In vitro, CSPG4‐KO cells exhibited significantly reduced proliferation across all models (Figure 2D), with cell‐cycle analysis showing decreased G2‐M phase occupancy and proliferation index (Figure S2A). In contrast, wound‐healing and transwell assays demonstrated that migration and invasion capabilities remained largely unchanged following CSPG4 depletion (Figure S2B,C). In vivo, intravenous injection of wild‐type (WT) and CSPG4‐KO CNE‐2 cells into immunodeficient mice showed prolonged survival in the KO group, indicating reduced systemic metastatic potential (Figure S2D).

To confirm the specificity of this oncogenic effect, we stably reconstituted CSPG4 expression in CSPG4‐KO CNE‐2 cells. Western blot and flow cytometry confirmed successful reconstitution of the target (Figure 2E,F). Notably, CSPG4 rescue restored in vitro proliferation rates and the proliferation index (Figure 2G,H). In vivo, CSPG4‑KO CNE‑2 cells exhibited a markedly reduced capacity to form subcutaneous tumors, whereas re‑expression of CSPG4 fully restored their tumorigenicity, underscoring that CSPG4 is a primary driver of HNSCC cell proliferation both in vitro and in vivo (Figure 2I–K).

To dissect the functional interplay between CSPG4 and CD44, we generated a series of isogenic CNE‐2 cell models: CSPG4‐KO, CD44 knockdown (KD) using shRNA, and dual‐ablated CSPG4‐KO + CD44 KD cells. Flow cytometric analysis verified the successful ablation of the respective targets (Figure 2L). Functional characterization of these models demonstrated that while knockout of CSPG4 or knockdown of CD44 significantly reduced cell proliferation and colony‐forming capacity, concomitant ablation of both CSPG4 and CD44 resulted in a synergistic inhibitory effect (Figure 2M,N; Figure S2E). This suggests a cooperative role in promoting tumor cell fitness. Given CD44's established role as a CSC marker, we evaluated oncosphere formation capability. While CD44 KD significantly impaired the ability of CNE‐2 cells to form spheres, the combined depletion of CSPG4 and CD44 further exacerbated this impairment (Figure 2O), indicating that CSPG4 is essential for maintaining the CSC‐like properties of HNSCC. Consistent with a stemness‐driven resistance mechanism, CD44 knockdown sensitized cells to cisplatin, an effect that was significantly potentiated by CSPG4 knockout (Figure 2P). Collectively, these findings validate that CSPG4 and CD44 functionally cooperate to drive the aggressive progression, stemness, and therapeutic resistance of HNSCC. =

### 2.3 CSPG4 Correlates with an Immunosuppressive Microenvironment and Oncogenic Programs in HNSCC

**FIGURE 3:**
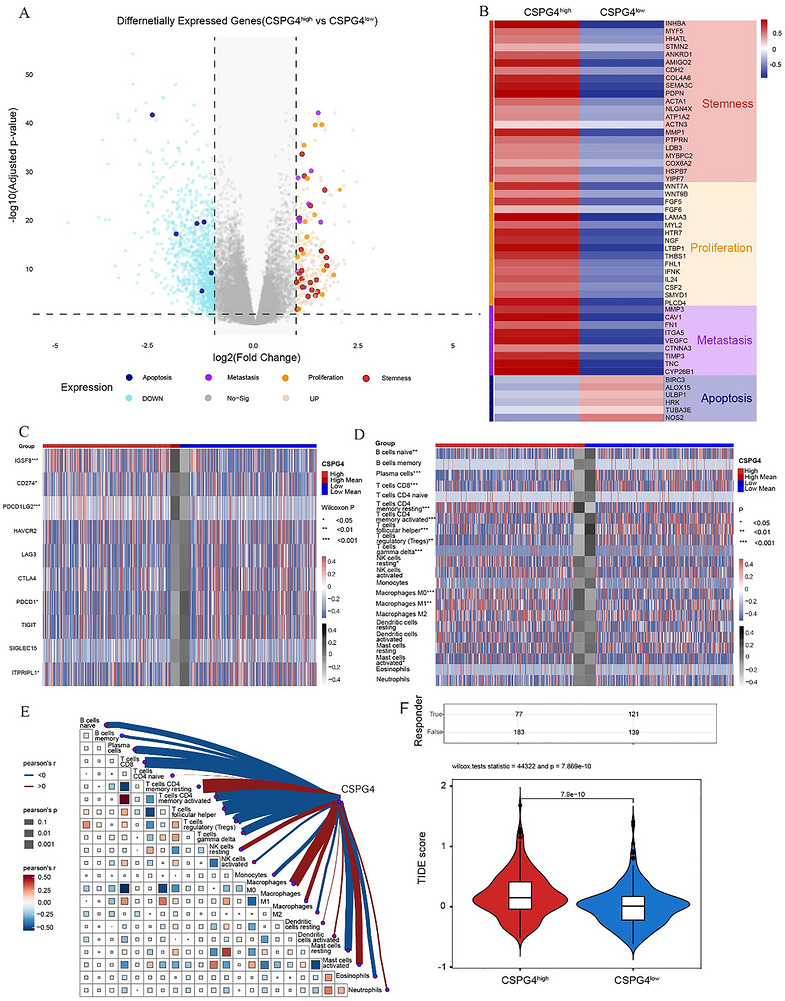
Transcriptional and immune landscape of CSPG4^high^ vs. CSPG4^low^ HNSCC. (A) Volcano plot of differentially expressed genes between TCGA HNSCC tumors stratified by high and low CSPG4 mRNA expression. (B) Heatmap illustrating the expression of gene signatures associated with stemness, proliferation, metastasis, and apoptosis. (C) Heatmap of immune checkpoint–related transcripts in CSPG4^high^ and CSPG4^low^ cohorts. (D) CIBERSORT deconvolution analysis comparing the relative abundance of 22 immune cell subsets in CSPG4^high^ vs. CSPG4^low^ tumors. (E) Correlation analysis between CSPG4 expression and various immune cell infiltrates. (F) Comparison of TIDE scores (bottom) and predicted immunotherapy responder proportions (top) between CSPG4^high^ and CSPG4^low^ groups. Error bars represent Mean ± SEM (^*^ *p* < 0.05, ^**^ *p* < 0.01, ^***^ *p* < 0.001).

To elucidate the molecular mechanisms and immunological correlates of CSPG4 elevation, we stratified TCGA HNSCC tumors into CSPG4‐high (CSPG4^high^) and ‐low (CSPG4^low^) cohorts. A volcano plot of differentially expressed genes (DEGs) identified a distinct transcriptomic signature associated with high CSPG4 expression (Figure 3A). Functional annotation further revealed that CSPG4^high^ tumors were enriched for gene sets involved in proliferation, metastasis, and stemness, whereas apoptosis‐related genes were down‐regulated (Figure 3B).

Given that immune checkpoint blockade (ICB) targeting the PD‐1/PD‐L1 axis is a standard of care for recurrent/metastatic HNSCC [18, 19], we compared immune‐related features between CSPG4^high^ and CSPG4^low^ tumors. Examination of immune checkpoints showed that IGSF8, PD‐L1, and PD‐L2 transcripts were significantly elevated in the CSPG4^high^ group (Figure 3C), suggesting that CSPG4^high^ HNSCC may harbor a more immunosuppressive tumor microenvironment. To profile tumor immune composition, we applied CIBERSORT, a validated computational deconvolution algorithm that estimates the relative proportions of immune cell subsets from bulk RNA‐seq data [20]. Using CIBERSORT, CSPG4^high^ tumors displayed reduced infiltration of naïve B cells, plasma cells, CD8^+^ T cells, activated memory CD4^+^ T cells, follicular helper T cells (T_fh_), and γδ T cells, together with increased proportions of resting memory CD4^+^ T cells, resting NK cells, and both M0 and M1 macrophages (Figure 3D). Correlation analysis confirmed a strong negative association between CSPG4 expression and both CD8^+^ T cells and T_fh_, immune subsets that are linked to improved outcomes after PD‐1 blockade (Figure 3E) [21]. Conversely, CSPG4^high^ tumors were enriched for resting memory CD4^+^ T cells and non‐polarized (M0) macrophages, a composition characteristic of a less‐inflamed, more immunosuppressive tumor microenvironment (Figure 3E) [22, 23].

To evaluate the potential for immune evasion, we employed the Tumor Immune Dysfunction and Exclusion (TIDE) framework [24]. TIDE integrates signatures of T cell dysfunction and exclusion, and higher scores predict a lower probability of benefit from PD‐1/PD‐L1 blockade [25]. CSPG4^high^ tumors exhibited significantly elevated TIDE scores compared to CSPG4^low^ tumors (Figure 3F), indicating a higher likelihood of T cell dysfunction and immune exclusion. These findings suggest that patients with elevated CSPG4 are less likely to respond to PD‐1‐based therapy and may require alternative immunotherapeutic interventions.

To validate these bioinformatic findings in vitro, we performed targeted qPCR profiling in CNE‐2, 5–8F, and 6–10B cells following CSPG4 knockout, which confirmed a broad downregulation of transcripts linked to stemness, proliferation, chromatin regulation, invasion, metabolism, and immune response (Figure S3A). To further define CSPG4‐dependent programs, we performed bulk RNA‐seq on WT, CSPG4‐KO, and Rescue CNE‐2 cells. Group‐wise analysis showed coordinated downregulation of transcripts linked to stemness, proliferation, and metastasis and upregulation of apoptosis‐related genes after CSPG4 loss, phenotypes that were largely reversed by CSPG4 re‐expression (Figure S3B). Gene ontology (GO) analysis of the DEGs in CSPG4‐KO cells further implicated RNA binding, metabolic processes, organelle and nuclear functions, and enzymatic activity (Figure S3C). Together, these data indicate that CSPG4 coordinates an extensive oncogenic network that supports HNSCC progression and immune recalcitrance.

### 2.4 CSPG4‑Directed CAR‑T Cells Exhibit HNSCC Cytotoxicity yet Suffer Excessive Tonic Signaling

**FIGURE 4:**
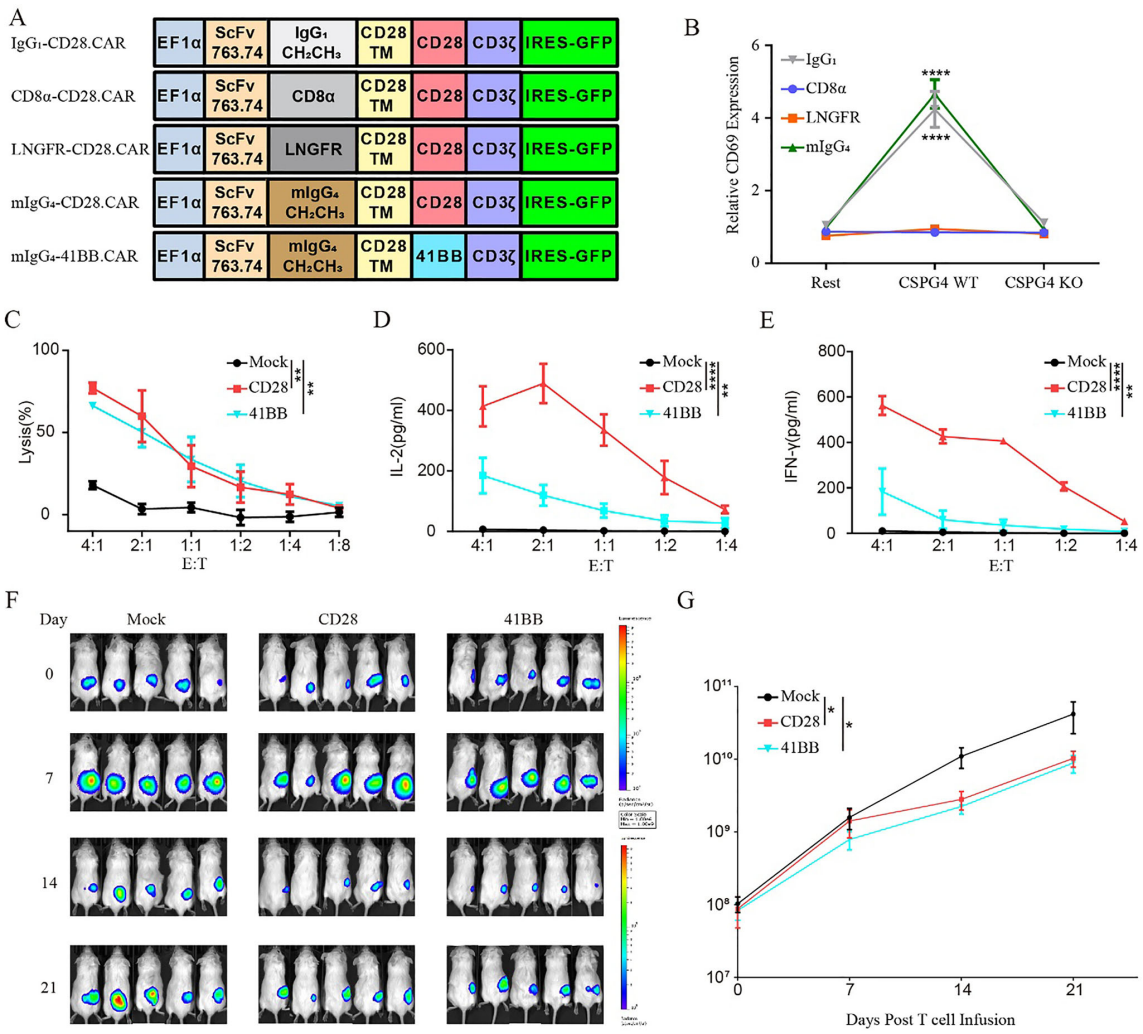
Hinge optimization and functional characterization of CSPG4.CAR‐T cells. (A) Schematic of CAR design utilizing the murine 763.74 scFv with four distinct hinge variants and CD28 or 4‐1BB costimulatory domains. (B) Relative CD69 expression on CAR‐T cells bearing different hinges following co‐culture with WT or CSPG4‐KO CNE‐2 cells. (C) In vitro cytotoxicity of mIgG_4_‐hinge CAR‐T cells with CD28 or 4‐1BB domain against CNE‐2 targets at the indicated E:T ratios. (D‐E) Secretion of IL‐2 (D) and IFN‐γ (E) by CSPG4.CAR‐T cells following tumor cell engagement. (F‐G) Representative bioluminescence imaging (F) and quantitative tumor growth curves (G) of NSG mice bearing subcutaneous CNE‐2 tumors treated with the indicated CAR‐T cells (n = 5 per group). Error bars represent Mean ± SEM (^*^ *p* < 0.05, ^**^ *p* < 0.01, ^****^ *p* < 0.0001).

To evaluate the therapeutic potential of targeting CSPG4, we constructed CSPG4‐specific CARs using a scFv derived from the murine monoclonal antibody 763.74 [26]. A commonly used spacer in CAR design includes an IgG_1_ hinge and the CH_2_‐CH_3_ Fc domain. However, previous studies have shown that this IgG_1_ Fc spacer can lead to unintended activation of CAR‐T cells by Fcγ receptor (FcγR)‐expressing cells, along with off‐target cytokine release from innate immune cells in vitro [27]. To mitigate these effects, we tested three alternative hinge variants incorporating CD8α, LNGFR, or a mutated IgG_4_ CH_2_‐CH_3_ (mIgG_4_) spacer that does not engage FcγRs (Figure 4A) [28, 29, 30]. Following co‐culture with WT and CSPG4‐KO CNE‐2 cells, only the CAR‐T constructs containing the mIgG_4_ spacer upregulated the activation marker CD69 in a CSPG4‐dependent manner (Figure 4B), indicating that only CAR‐T cells bearing this spacer mediate specific recognition of CSPG4 on target cells.

Subsequent investigations focused on CAR constructs incorporating this mIgG_4_ hinge. CAR surface expression was confirmed by flow cytometry in T cells engineered with either CD28 or 4‐1BB costimulatory domains (Figure S4A), with both constructs showing comparable transduction efficiencies (Figure S4B). In vitro cytotoxicity assays demonstrated that both CSPG4.CD28.CAR‐T and CSPG4.4‐1BB.CAR‐T cells effectively lysed HNSCC target cells with similar potency (Figure 4C). However, upon antigen engagement, CSPG4.CD28.CAR‐T cells secreted significantly higher levels of IL‐2 and IFN‐γ compared to their 4‐1BB counterparts, indicating a more robust functional activation (Figure 4D,E). In vivo assessment in xenograft models demonstrated that both CSPG4.CAR‑T constructs modestly delayed tumor progression, as monitored by bioluminescence imaging (Figure 4F). Quantitative imaging analysis confirmed limited yet statistically significant tumor suppression in both treatment groups (Figure 4G).

Given that tonic signaling can drive spontaneous CAR‐T cell exhaustion and impair antitumor function [31], we next evaluated the tonic signaling strength of CSPG4.CAR‐T cells. Under resting, antigen‑free conditions, cytokine profiling revealed that both CSPG4.CD28.CAR‐T and CSPG4.4‐1BB.CAR‐T cells secreted elevated levels of proinflammatory cytokines, with the CD28‑based construct exhibiting the highest spontaneous release (Figure S4C). Flow cytometric analysis further demonstrated increased expression of activation markers and exhaustion markers on both CD8^+^ and CD4^+^ CAR‐T cell subsets compared to untransduced T cells (Figure S4D,E), indicating that CSPG4.CAR‐T cells display significant tonic signaling and pronounced exhaustion even in the resting state. Together, these data suggest that, although CSPG4.CAR‐T cells effectively recognize and eliminate CSPG4‑positive HNSCC cells in, their therapeutic efficacy is likely constrained by excessive tonic signaling.

### 2.5 Humanization and Charge Optimization of CSPG4.CAR Mitigates Tonic Signaling and Exhaustion

**FIGURE 5:**
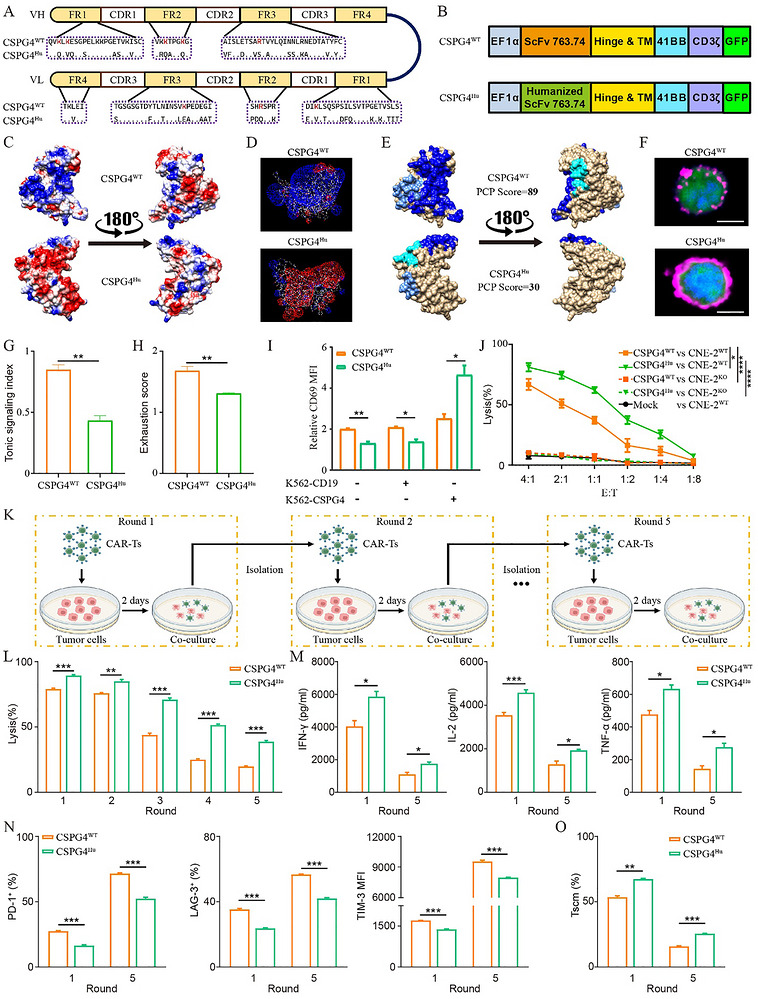
Humanization and charge optimization of CSPG4.CAR reduces tonic signaling and enhances durability. (A) Diagram of murine‐to‐human framework grafting in the 763.74 scFv to remove positive‐charge residues. (B) Structure of humanized CSPG4.CAR. (C) Surface charge residue mapping on wild‐type and humanized scFv (blue = positive, red = negative). (D) Electrostatic field distribution on CAR surfaces (blue = positive field, red = negative, area = field strength). (E) Quantification of the three largest positive‐charge patches (PCPs) on wild‐type vs. humanized CAR surfaces. (F) Immunofluorescence of CAR‐Jurkat cells showing clustering of wild‐type CAR vs. diffuse distribution of humanized CAR. (G‐H) Tonic signaling index (G) and Exhaustion score (H) comparison in resting wild‐type and humanized CAR‐T cells. (I) CD69 MFI on CAR‐T cells following stimulation with K562 or K562‐CSPG4 cells. (J) Specific lysis of wild‐type (CNE‐2^WT^) or isogenic CSPG4‐KO (CNE‐2^KO^) target cells by CSPG4^WT^ or CSPG4^Hu^ CAR‐T cells at the indicated effector‐to‐target (E:T) ratios after 24‐h co‐culture. (K) Schematic representation of the serial in vitro cytotoxicity and re‐stimulation assay. Briefly, 10^6^ luciferase‐expressing CNE‐2 cells were co‐cultured with CAR‐T cells at an E:T ratio of 1:1. Every 2 days, viable CAR‐T cells were counted and re‐introduced to fresh tumor cells at the same ratio for five consecutive rounds. (L‐M) Cumulative tumor lysis (L) and cytokine secretion (M) across the five rounds of challenge. (N) Frequency of exhaustion markers (PD‐1^+^, LAG‐3^+^) and TIM‐3 MFI across the five rounds of challenge. (O) Maintenance of the stem cell‐like memory T cell (Tscm​) subset during the serial challenge process. Error bars represent Mean ± SEM (^*^ *p* < 0.05, ^**^ *p* < 0.01,^***^ *p* < 0.001, ^****^ *p* < 0.0001).

To minimize immunogenicity and tonic signaling, we grafted the murine CDR of the 763.74 scFv onto a human antibody scaffold engineered with reduced positively charged residues (Figure 5A), yielding a humanized CSPG4.CAR (CSPG4^Hu^.CAR) (Figure 5B). Humanization scoring demonstrated a marked improvement in humanness [32], with the heavy‑chain H‑score increasing from −1.801 to −0.458 and the light‑chain H‑score improving from −2.340 to −0.987 (Figure S5A). Structural modeling revealed that, compared to the wild‑type CSPG4.CAR (CSPG4^WT^.CAR), CSPG4^Hu^.CAR exhibited markedly fewer surface positive‑charge residues (Figure 5C) and a markedly contracted positive electrostatic field (Figure 5D). Quantitative analysis of positively charge patches (PCPs), calculated as the combined surface area of the three largest PCPs, revealed that the PCP score decreased from 89 in CSPG4^WT^.CAR to 30 in CSPG4^Hu^.CAR (Figure 5E) [12]. These data indicated that our scFv optimization effectively reduced both the immunogenicity and the surface positively‐charged clusters of the CSPG4.CAR.

Next, we evaluated the impact of these modifications on CAR behavior. Flow cytometry confirmed equivalent surface expression of both constructs on Jurkat T cells (Figure S5B). When co‑cultured with CSPG4^+^ CNE‐2 targets, both CSPG4^WT^.CAR‐Jurkat and CSPG4^Hu^.CAR‐Jurkat cells specifically upregulated CD69, with no induction seen using CSPG4‑KO CNE‐2 cells (Figure S5C). Immunofluorescence of CAR‐Jurkat cells showed that CSPG4^WT^.CAR formed prominent surface clusters, whereas CSPG4^Hu^.CAR exhibited a diffuse membrane distribution, indicating that spontaneous oligomerization was effectively suppressed (Figure 5F). Consistent with this, in the absence of antigen, CSPG4^Hu^.CAR‑Jurkat cells expressed lower basal levels of CD69 and PD‑1compared to the WT group (Figure S5D,E).

In primary human T cells, Western blot using the anti‑phosphotyrosine antibody 4G10 showed that CSPG4^Hu^.CAR‑T cells had significantly lower basal CD3ζ ITAM phosphorylation compared to CSPG4^WT^.CAR‑T cells (Figure S5F). Furthermore, resting CSPG4^Hu^.CAR‐T cells exhibited diminished tonic signaling, evidenced by reduced surface levels of CD69, CD25, and ICOS (Figure S5G) and a significantly lower tonic signaling index (Figure 5G). Accordingly, these cells displayed decreased levels of exhaustion markers PD‑1, LAG‑3, and TIM‑3 (Figure S5H), resulting in a markedly lower exhaustion score than that of CSPG4^WT^.CAR‐T cells (Figure 5H).

Functional evaluation revealed that while CSPG4^Hu^.CAR‐T cells maintained quiescence under resting conditions, they displayed superior responsiveness upon target engagement. CSPG4^Hu^.CAR‐T cells showed significantly higher CD69 upregulation specifically after stimulation with K562‐CSPG4 cells compared to the CSPG4^WT^ group (Figure 5I). As shown in Figure 5J, CSPG4^Hu^.CAR‐T cells demonstrated significantly enhanced lytic potency against CNE‐2 cells compared to their WT counterparts. Importantly, both CARs exhibited minimal background killing against CSPG4‐KO targets, confirming that the enhanced cytotoxicity remained strictly antigen‐dependent.

To assess long‐term durability, we performed a serial re‐stimulation assay (Figure 5K). CSPG4^Hu^.CAR‐T cells demonstrated significantly higher tumor lysis rates and superior secretion of IFN‐γ, IL‐2, and TNF‐α across five consecutive rounds of challenge (Figure 5L,M). Phenotypic analysis following serial killing revealed that CSPG4^Hu^.CAR‐T cells were more resistant to exhaustion, maintaining lower frequencies of PD‐1^+^ and LAG‐3^+^ cells and reduced TIM‐3 expression (Figure 5N; Figure S6A–C). Crucially, CSPG4^Hu^.CAR‐T cells retained a significantly higher frequency of stem cell‐like memory T cells (T_scm_​) throughout the process (Figure 5O; Figure S6D). Together, these findings demonstrate that the CSPG4^Hu^.CAR design effectively suppresses tonic signaling and early exhaustion while enhancing long‐term persistence and potency.

### 2.6 CSPG4^Hu^.CAR‑T Cells Exhibit Enhanced Efficacy and Persistence in HNSCC Models

**FIGURE 6:**
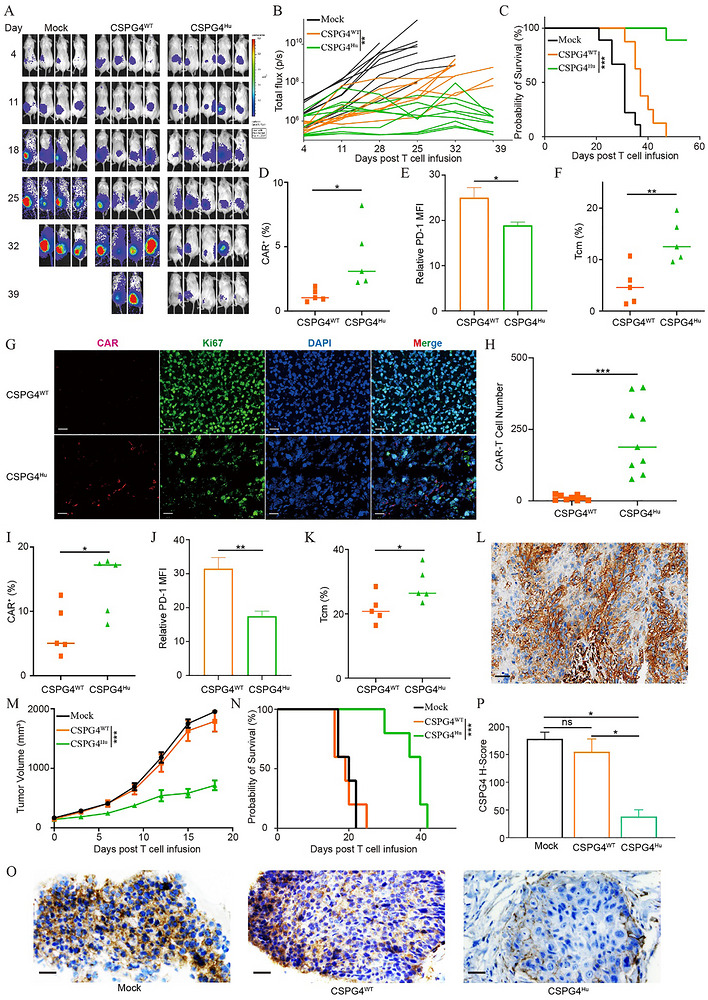
Humanized and charge‐optimized CSPG4.CAR‐T cells exhibit enhanced antitumor efficacy and persistence in vivo. (A–C) BLI imaging (A), tumor burden quantification (B), and survival curves (C) of CNE‐2‐bearing mice. D‐F) Frequency of CAR^+^ cells (D), PD‐1 MFI (E), and T_cm_​ percentage (F) in the peripheral blood of treated mice. (G‐H) Representative immunofluorescence images (G) and quantitative analysis (H) of CAR‐T cell (red) and Ki‐67 (green) levels within the TME. (I–K) Percentage of CAR^+^ TILs (I), PD‐1 expression (J), and T_cm_​ differentiation (K) within the tumor. (L–N) CSPG4 IHC of PDX tumors (L), tumor volume growth curves (M), and Kaplan–Meier survival analysis (N) in the PDX model. (O‐P) Representative IHC images (O) and H‐score quantification (P) of CSPG4 expression in tumors at the experimental endpoint. Error bars represent Mean ± SEM (^*^ *p* < 0.05, ^**^ *p* < 0.01, ^***^ *p* < 0.001).

Building on the in vitro demonstration that humanization and charge reduction attenuate antigen‐independent tonic signaling and delay exhaustion, we evaluated whether these molecular and phenotypic changes translate to superior antitumor activity in vivo. In a luciferase‐expressing CNE‐2 xenograft model, longitudinal bioluminescence imaging showed that CSPG4^Hu^.CAR‐T induced rapid, durable tumor regression, whereas CSPG4^WT^.CAR‐T produced only modest, transient control (Figure 6A,B). Concordantly, mice treated with CSPG4^Hu^.CAR‐T experienced a significant survival advantage (Figure 6C).

Phenotypic analyses linked improved tumor control to enhanced CAR‐T persistence and preservation of a less‐exhausted differentiation state. In peripheral blood, CSPG4^Hu^.CAR‐T cells comprised a larger fraction of human CD8^+^ cells and expressed lower PD‐1 while showing enrichment for central‐memory (c) markers relative to CSPG4^WT^.CAR‐T (Figure 6D–F). Equivalent shifts toward reduced PD‐1 and increased T_cm_ frequencies were apparent within the CD4^+^ compartment (Figure S7A–I). Within the tumor microenvironment, quantitative immunofluorescence and flow cytometry revealed greater infiltration of mCherry^+^ CAR‐T cells, decreased tumor Ki‐67, and a higher proportion of CD8^+^ TILs with low PD‐1 and a T_cm_ phenotype in CSPG4^Hu^.CAR‐T–treated tumors (Figure 6G–K; Figure S7J–M).

To assess clinical relevance, we tested both constructs in an HNSCC PDX model. CSPG4^Hu^.CAR‐T markedly suppressed PDX growth and prolonged survival compared with CSPG4^WT^.CAR‐T and mock controls (Figure 6L–N). Body weights remained comparable across groups, indicating no overt toxicity (Figure S7O). To assess the antigen‐specific impact of CAR‐T cell therapy in vivo, we performed immunohistochemical staining for CSPG4 on PDX tumor sections collected at the endpoint. Quantitative analysis revealed a decrease in CSPG4 H‐Scores within tumors treated with either CSPG4^WT^ or CSPG4^Hu^ CAR‐T cells compared to mock‐treated controls (Figure 6O,P). Notably, the most substantial reduction was observed in the CSPG4^Hu^ group, correlating with its superior therapeutic efficacy. This decrease in target antigen density directly reflects the effective elimination of CSPG4‐positive tumor cells in situ and serves as concrete histological evidence of successful, antigen‐specific tumor targeting by our CAR‐T cells.

### 2.7 Transcriptomic Profiling Reveals Enhanced Fitness and Stem‐Like Signature in Optimized CSPG4^Hu^ CAR‐T Cells

**FIGURE 7:**
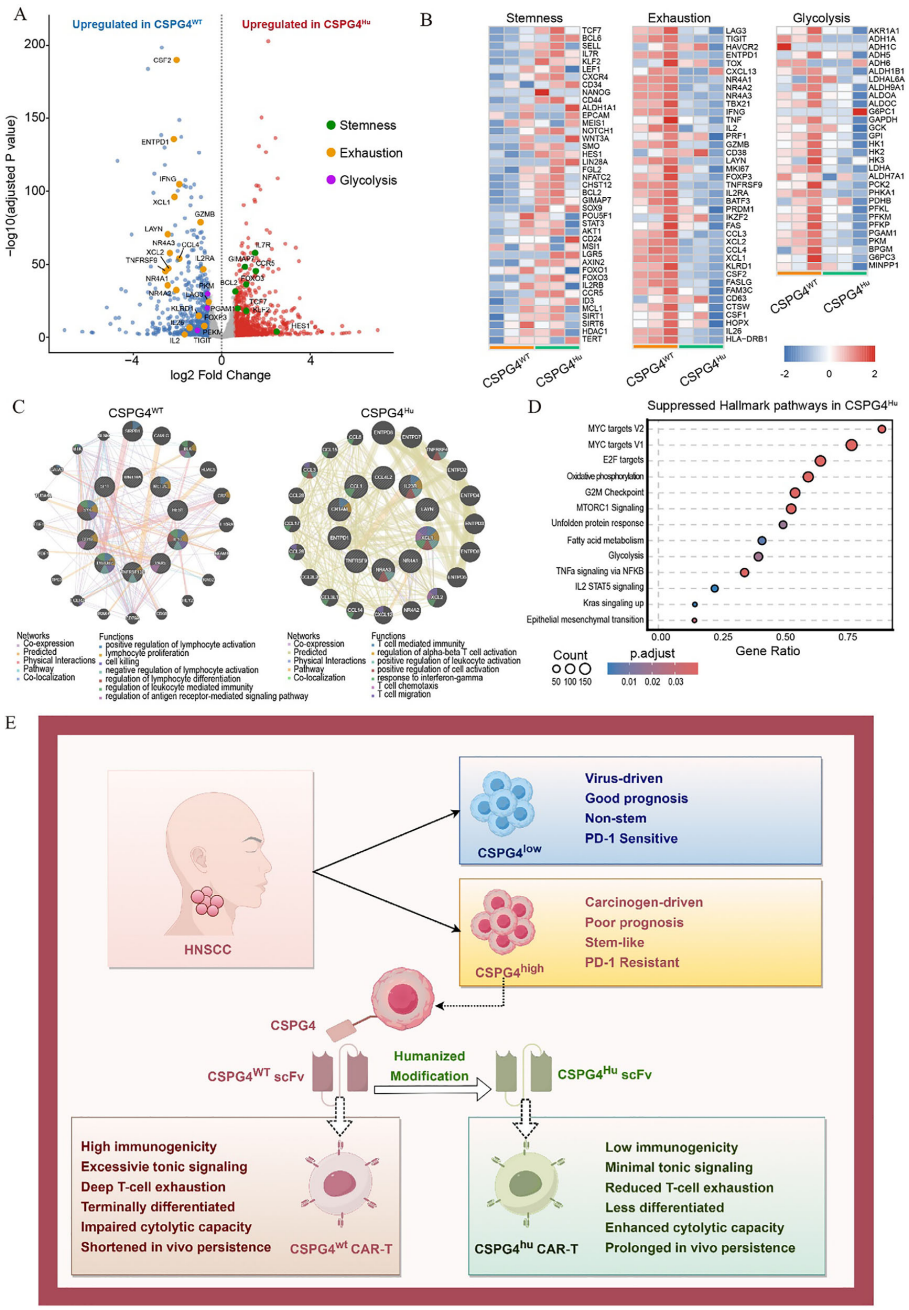
Transcriptomic profiling and conceptual model of humanized CSPG4.CAR‐T cell superiority. (A) Volcano plot of DEGs between CSPG4^WT^ and CSPG4^Hu^ CAR‐T cells. (B) Representative heatmaps comparing the expression profiles of gene sets related to stemness, exhaustion, and glycolysis. (C) Functional interaction networks of DEGs in CSPG4^WT^ and CSPG4^Hu^ CAR‐T cells. (D) Dot plot of suppressed Hallmark pathways in CSPG4^Hu^ CAR‐T cells. (E) Overall conceptual model. High CSPG4 expression in HNSCC correlates with a carcinogen‐driven, CSC‐like, and PD‐1 resistant phenotype with poor prognosis. While CSPG4^WT^ CAR‐T cells suffer from high immunogenicity, excessive tonic signaling, and deep exhaustion, the humanized modification effectively yields a CAR‐T product with low immunogenicity, minimal tonic signaling, and superior persistence, leading to enhanced therapeutic efficacy in HNSCC.

To elucidate the molecular mechanisms underlying the superior performance of CSPG4^Hu^ CAR‐T cells, we performed comprehensive RNA‐seq to compare the transcriptomic landscapes of CSPG4^WT^ and CSPG4^Hu^ cells. Principal component analysis (PCA) revealed distinct spatial separation between the two groups, indicating a profound transcriptomic shift following humanization and charge optimization (Figure S8A). Volcano plot analysis identified a significant number of DEGs, with key exhaustion markers enriched in the CSPG4^WT^ group and stemness‐associated genes upregulated in the CSPG4^Hu^ group (Figure 7A). Detailed heatmap and single‐sample gene set enrichment analysis (ssGSEA) further characterized these phenotypic differences. CSPG4^Hu^ CAR‐T cells exhibited a robust stem‐like signature, characterized by the high expression of TCF7, LEF1, SELL, and IL7R (Figure 7B; Figure S8B). In stark contrast, CSPG4^WT^ cells were dominated by a terminal exhaustion profile, with significant elevation of LAG3, TIGIT, TOX, NR4A1, NR4A2, and NR4A3. Furthermore, CSPG4^WT^ cells showed a marked enrichment in glycolytic enzymes (e.g., LDHA, PKM, PGAM1), whereas CSPG4^Hu^ cells maintained a significantly lower glycolytic ssGSEA score, suggesting reduced metabolic stress and preserved bioenergetic flexibility in CSPG4^Hu^ cells.

GeneMANIA network analysis underscored the functional divergence. CSPG4^WT^ cells displayed a network centered on chronic activation and exhaustion hubs, while CSPG4^Hu^ cells were associated with pathways regulating T cell differentiation and migration (Figure 7C). To identify the specific signaling pathways driving these phenotypes, we performed Hallmark GSEA. The analysis revealed that CSPG4^Hu^ cells significantly suppressed pathways related to proliferative stress and metabolic overdrive, including MYC targets (V1/V2), E2F targets, oxidative phosphorylation, and glycolysis (Figure 7D). Finally, GO and KEGG enrichment analyses confirmed that while CSPG4^WT^ cells were primarily involved in stress responses and inflammatory signaling, CSPG4^Hu^ cells were enriched in genes facilitating lymphocyte differentiation and PI3K/Akt signaling (Figure S8C,D). Together, these findings show that CAR humanization and charge optimization reprogram CAR‐T cells away from tonic signaling‐induced exhaustion and metabolic overactivation toward a stem‐like, memory‐associated transcriptional state that underlies enhanced persistence.

Figure 7E integrates our findings and highlights a coherent biological context. CSPG4‐positive HNSCCs are enriched among HPV‐negative, chemically associated tumors and display CSC‐like features together with an immunosuppressive tumor microenvironment, a combination that correlates with resistance to PD‐1 blockade and poorer clinical outcomes. CARs built on the original murine 763.74 scFv showed high predicted immunogenicity, prominent tonic signaling, and a terminally exhausted, over‐differentiated transcriptional and phenotypic program, which was associated with reduced cytotoxicity and limited in vivo persistence. In contrast, humanization combined with surface charge optimization substantially reduced immunogenicity and positively‐charged clustering, attenuated tonic signaling, lowered exhaustion marker expression, and preserved a stem‐/memory‐biased differentiation state. These improvements correlated with more potent antigen‐specific killing and markedly enhanced durability in multiple HNSCC models.

## 3 Discussion

Our findings identify CSPG4 as a highly expressed antigen in advanced, HPV‐negative HNSCC and a marker of poor patient outcome, with broad implications for understanding tumor biology and developing new therapies. This observation aligns with the established prognostic role of CSPG4 in HNSCC. Previous studies have demonstrated that its overexpression, driven by promoter hypomethylation, correlates with poor survival [33], and that its de novo expression independently predicts loco‐regional relapse [34]. The role of CSPG4 as a marker for aggressive HNSCC is not confined to this specific cancer but extends to other squamous cell carcinomas and, more broadly, to a range of other solid tumors [35]. For example, CSPG4 is overexpressed in the majority of glioblastomas and soft‐tissue sarcomas [36, 37, 38], and CSPG4‐directed CAR‐T cells control these tumors in vitro and in vivo [39, 40, 41]. Notably, CSPG4 is upregulated by inflammatory signals and is expressed on tumor neurospheres with stem‐cell‐like properties [11, 42]. Our clinical data extend these observations to HNSCC, suggesting that CSPG4 is a biomarker of a chemically induced, HPV‐negative subtype that behaves like a CSC‐like, treatment‐refractory malignancy. Importantly, higher CSPG4 levels were statistically linked to recurrence and malignant transformation in a vocal‐fold leukoplakia cohort, reinforcing its role as a prognostic indicator [43]. Taken together, these findings establish CSPG4 as a pan‐cancer onco‐antigen whose enrichment in HPV‐negative HNSCC identifies tumors with worse outcomes and CSC‐like, invasive characteristics.

HNSCC transcriptional profiling revealed a CSPG4^high^ tumor subpopulation co‐expressing CD44 and enriched in stemness‐ and EMT‐related gene signatures, implicating CSPG4 in an aggressive phenotype. This observation is consistent with broad evidence that CSPG4 acts as an oncoantigen linking extracellular‐matrix signals to key oncogenic pathways [44, 45]. In the tumour microenvironment, CSPG4 engages integrin complexes to activate FAK/Src and PI3K/AKT signaling and to sustain MAPK/ERK activity [46]. These cascades underlie CSPG4‐driven proliferation, survival, and motility: for example, CSPG4 overexpression prolongs ERK1/2 phosphorylation, induces fibronectin and other mesenchymal markers, and suppresses E‐cadherin, driving EMT [44]. In ovarian carcinoma, CSPG4 likewise elevates FAK signaling and the EMT transcription factor ZEB1 to promote invasion, spheroid formation, and cisplatin resistance [47]. Consistently, CSPG4 knockdown in chondrosarcoma cells markedly reduces proliferation, migration, and protease expression and sensitizes tumors to doxorubicin [48], whereas CSPG4‐positive progenitor‐like glioblastoma cells exhibit enhanced DNA‐damage recognition, ROS scavenging and checkpoint signaling that confer radioresistance [49]. Notably, Macaulay et al. showed that in RDEB‐associated cutaneous SCC, CSPG4 localizes to invasive tumor fronts and potentiates membrane‐proximal TGF‐β/SMAD3 signaling to drive EMT and invasion [50], mirroring the invasive‐EMT signatures we observe in CSPG4^high^ HNSCC cells. Moreover, chondroitin‐sulfate‐modified CSPG4 creates a pericellular glyco‐niche that maintains glioma‐initiating cell stemness by reinforcing integrin‐ERK signaling and blocking differentiation cues [42]. In HNSCC, CSPG4^high^ cells co‐express CD44, a CSC marker that mediates PI3K‐4EBP1‐SOX2 signaling in response to hyaluronan [17], suggesting CSPG4 may cooperate with CD44 to amplify stemness circuits. Finally, in BRAF‐mutant thyroid cancer, CSPG4 mediates adaptive resistance to MAPK inhibitors by sustaining feedback activation of multiple receptor tyrosine kinases, and its loss abrogates this RTK feedback to restore BRAF inhibitor sensitivity [51]. Taken together, these prior studies and our data converge on a unified model in which CSPG4 functions as a membrane scaffold that coordinates integrin/FAK, MAPK/ERK, PI3K/AKT, and TGF‐β signaling to drive CSC‐like programs, invasive behavior, and therapeutic resistance in solid tumors, suggestting that targeting CSPG4 could simultaneously attack bulk tumor cells and the tumor‐initiating population.

HNSCC with high CSPG4 expression exhibits features of a more immunosuppressive tumor microenvironment. Specifically, these tumors display elevated expression of immune checkpoint molecules such as IGSF8, PD‐L1, and PD‐L2. IGSF8 has recently been identified as an innate immune checkpoint, which binds inhibitory receptors on NK cells and suppresses NK cell‐mediated cytotoxicity [52]. In contrast, PD‐L1 and PD‐L2 are well‐known ligands for the T‐cell checkpoint receptor PD‐1, the overexpression of which could dampen T‐cell activation and promote tumor immune evasion [53]. Using the CIBERSORT deconvolution algorithm, transcriptomic profiling revealed that CSPG4^high^ tumors were depleted of antitumor effector immune subsets, including CD8^+^ T cells and T_fh_. Prior studies have demonstrated that robust infiltration of CD8^+^ T cells and T_fh_ cells is associated with improved outcomes following immune checkpoint blockade [54], whereas the immune composition observed in CSPG4^high^ tumors lacks these favorable prognostic features. TIDE is a rigorously validated framework for predicting response to immune checkpoint blockade [55]. Using the TIDE algorithm, we found that CSPG4^high^ tumors exhibit a greater degree of immune dysfunction and exclusion, suggesting that patients with elevated CSPG4 expression are less likely to derive benefit from PD‐1‐based therapies. Taken together, these findings indicate that CSPG4^high^ HNSCCs are characterized by a low‐inflammation, highly immunosuppressive microenvironment, implying poor responsiveness to PD‐1 blockade and underscoring the need for more effective immunotherapeutic strategies tailored for this subgroup of patients.

Given CSPG4's role in aggressive and immunosuppressive HNSCC, our study explored CSPG4‐directed CAR‐T therapy. We confirm that a murine anti‐CSPG4 scFv can direct CAR‐T killing of CSPG4‐positive HNSCC cells in vitro and modestly delay tumor growth in vivo, but we also reveal that this first‐generation design suffers from two key problems: high immunogenicity and excessive tonic signaling. High immunogenicity of murine scFv‐based CARs accelerates clearance of the therapeutic cells and can provoke anti‐CAR humoral and cellular responses in the host, thereby shortening persistence and limiting repeated dosing [56, 57]. Strategies to mitigate immunogenicity center on replacing non‐human sequence elements with human counterparts. Common approaches include complementarity determining region (CDR) grafting onto human germline frameworks, in‐silico deimmunization to remove predicted T‐cell epitopes, selection of fully human binders from phage or yeast libraries, and careful codon optimization to avoid neoepitopes [58]. These methods reduce the likelihood of anti‐drug responses and thereby improve CAR‐T persistence and the feasibility of repeated administration.

Excessive tonic signaling is an independent and equally important barrier [59]. Antigen‐independent CAR clustering produces basal CD3ζ ITAM phosphorylation, spontaneous cytokine release, premature upregulation of activation and exhaustion markers, and accelerated differentiation toward short‐lived effector phenotypes [12, 60]. Multiple engineering solutions have been described to temper tonic signaling, including modulating scFv affinity or stability [61, 62], shortening the scFv linker [63], replacing costimulatory modules [60], and using different CD3 subunits [64]. Our previous work has demonstrated that positively charged patches on the CAR surface are a major driver of excessive tonic signaling [12, 65]. In this study, we further developed a novel strategy by replacing the murine framework with a humanized scaffold optimized for electrostatic balance, thereby reducing both the immunogenicity and tonic signaling of CAR‐T cells and markedly enhancing the efficiency of CAR‐T optimization.

A central finding of our study is that the biophysical optimization of the CAR scFv fundamentally reprograms the T cell transcriptional and metabolic landscape, shifting its fate from terminal exhaustion to a stem‐like, memory‐associated state. In CSPG4^WT^ CAR‐T cells, the highly charged CAR architecture drives a state of persistent NFAT signaling, leading to the direct upregulation of NR4A family and TOX, which aligns precisely with the molecular signature of terminally exhausted T cells [66]. This state is coupled with a metabolic overdrive characterized by excessive glycolysis and MYC‐driven proliferative stress, which is a hallmark of CAR‐T failure in solid tumors [67]. In stark contrast, charge and immunogenicity optimization in CSPG4^Hu^ cells redirects them toward a stem‐like transcriptional program and metabolic rejuvenation. The upregulation of key transcription factors TCF1 and LEF1, alongside increased expression of the lymph node homing receptor SELL and the survival cytokine receptor IL7R, defines a stem‐like memory progenitor exhausted phenotype [68]. The enrichment of PI3K/Akt signaling further supports T cell survival and metabolic fitness [69]. Notably, CSPG4^Hu^ cells actively suppress the MYC, E2F, and mTORC1‐mediated metabolic stress pathways observed in the WT group, indicating a shift from a stressed, rapidly‐dividing state to a more quiescent yet bioenergetically flexible one [70]. This preserved fitness reservoir is likely indispensable for sustaining CAR‐T cell infiltration and effector functions within the immunosuppressive HNSCC microenvironment.

The selection of CSPG4 as a therapeutic target addresses several critical limitations inherent in other prominent solid‐tumor CAR antigens. While EGFRvIII is highly tumor‐specific, its clinical utility is frequently hampered by significant intratumoral heterogeneity and antigen loss under therapeutic pressure, leading to rapid antigen‐negative escape and relapse [71]. Pan‐ErbB‐directed CAR‐T therapy for HNSCC necessitated intratumoral administration to mitigate on‐target off‐tumor toxicity, yet failed to elicit any complete or partial clinical responses [72]. Similarly, MUC1‐directed CAR‐T therapies are constrained by potential on‐target off‐tumor toxicities, and the clinical efficacy is further hindered by the tumor's ability to mask critical MUC1 epitopes through aberrant glycosylation or conformational changes [73]. B7‐H3, despite its broad coverage, functions as an innate immune checkpoint ligand that can intrinsically suppress T‐cell metabolic fitness and effector function, potentially compromising the potency of the CAR‐T product from within [74]. In contrast, CSPG4 stands out due to its exceptionally high and homogeneous expression in HPV‐negative HNSCC, significantly reducing the likelihood of antigen escape. The translational feasibility and reliability of this target are further validated by the ongoing Phase I clinical trial (NCT06096038) evaluating CSPG4‐directed CAR‐T cells in HNSCC [75]. Critically, our humanized and charge‐optimized CAR design directly addresses the pivotal clinical barrier of T cell exhaustion by reprogramming cells toward a stem‐like memory phenotype, thereby mitigating the premature functional decline that commonly limits CAR‐T persistence in solid tumors.

A limitation of the present study is the exclusive use of severely immunodeficient NSG mice for our xenograft and PDX experiments. NSG mice lack mature T and B lymphocytes, have extremely low NK cell activity, and display multiple defects of innate immunity. Therefore, this model cannot assess critical interactions between the administered CAR‐T cells and the host's endogenous immune components. Second, while our in vitro co‐culture assays demonstrated excellent antigen specificity, a comprehensive safety profile against a broad panel of vital primary human normal tissues was beyond the scope of this work, and future dedicated toxicology studies are essential. Finally, our findings are most applicable to the CSPG4^high^, HPV‐negative HNSCC population, and the generalizability to HPV‐positive tumors requires further investigation. Addressing these limitations in future studies, particularly by validating our findings in fully humanized or autologous immune models, will be a crucial prerequisite for clinical translation.

## 4 Conclusion

This study advances the field of solid tumor immunotherapy by moving beyond modular CAR engineering toward a more integrated, biology‐driven therapeutic design. While humanization and electrostatic optimization have been explored in various contexts, our work demonstrates that their rational integration, specifically by selecting human germline frameworks with inherently favorable charge distributions, represents a superior approach to silencing scFv‐mediated tonic noise. More importantly, we identified CSPG4^high^ tumors as a specific, immunosuppressive niche that is intrinsically resistant to current checkpoint inhibitors. By bridging this gap with a structurally optimized, persistence‐enhanced CAR‐T product, our study provides a definitive roadmap for the clinical translation of humanized CSPG4.CAR‐T therapy. This approach moves the field toward precision immunotherapy, where the structural design of the synthetic receptor is meticulously tailored to the specific biological and metabolic demands of the target patient population.

## 5 Experimental Section/Methods

### 5.1 Ethics Approval for Human Samples

All procedures involving human participants complied with the Declaration of Helsinki and institutional guidelines. Written informed consent was obtained from all participants prior to sample collection. All surgical specimens and PBMCs were processed under the supervision of the Ethics Committee of the Eye & ENT Hospital of Fudan University (No. 2021155‐1).

### 5.2 Cell Lines and Reagents

HEK‐293T, Jurkat, and the panel of human HNSCC cell lines used in this study (FaDu, Cal‐27, HN6, HN‐8, HEp‐2, CNE‐1, CNE‐2, HNE‐2, 5–8F, 6–10B) as well as the immortalized nasopharyngeal epithelial control line NP69 were obtained from the American Type Culture Collection (ATCC) or the Chinese National Cell Bank. All cell lines were authenticated by the supplying repositories at the time of acquisition and were routinely tested and confirmed mycoplasma‐negative. Adherent HNSCC cell lines, NP69 and HEK‐293T packaging cells were maintained in Dulbecco's Modified Eagle Medium (DMEM) supplemented with 10% (v/v) fetal bovine serum (FBS) and 1% (v/v) penicillin–streptomycin. Jurkat cells were maintained in RPMI‐1640 medium supplemented with 10% (v/v) FBS and 1% (v/v) penicillin–streptomycin. All cells were cultured at 37°C in a humidified incubator with 5% CO_2_. Cells were used for experiments within 20 passages of thawing and were routinely monitored for morphology and growth rate. To ensure assay‐specific reliability, we systematically screened multiple commercially available anti‐CSPG4 antibodies across different applications. The specificity of each candidate antibody was rigorously validated using isogenic CNE‐2 cell models as negative and positive controls (Figure S9). Details of all reagents used are provided in Table S1.

### 5.3 TCGA and Bioinformatic Analyses

TCGA HNSCC RNA‐seq data were downloaded and processed using standard pipelines in R. Samples were stratified by CSPG4 mRNA expression into high and low groups (median split unless otherwise specified). Differential expression analysis was performed using DESeq2 [76]. Volcano plots, GO enrichment, and pathway annotations were generated using clusterProfiler [77]. Immune deconvolution was performed with CIBERSORT using the LM22 signature [20]. TIDE scores were computed using the TIDE web algorithm or local implementation [24]. Correlations and survival analyses (Kaplan–Meier, log‐rank) were carried out in R. Detailed parameter settings and R scripts are available upon request.

### 5.4 PCR

Fresh surgical specimens (tumor and matched adjacent mucosa) were collected and rinsed in ice‐cold PBS, snap‐frozen, and stored at −80°C. Total RNA was extracted from ∼20 to 30 mg tissue using TRIzol or column kits, quantified by NanoDrop/Qubit, and assessed for integrity (Bioanalyzer; RIN ≥7 when available). cDNA was synthesized from 0.5 to 1 µg RNA using standard reverse transcription kits. qPCR was performed using SYBR‐Green chemistry on a real‐time platform. Primer sequences are listed in Table S2. Amplification specificity was confirmed by melting‐curve analysis, and primer efficiency was validated by standard curves. Relative expression was calculated by the 2^−ΔΔCt^ method with GAPDH as the internal control. For HPV detection, DNA was extracted from tumor samples using a genomic DNA isolation kit, and PCR was performed using broad‐spectrum consensus primers targeting a wide range of HPV genotypes [78]. Amplicons were resolved by agarose gel electrophoresis, and representative positive products were confirmed by Sanger sequencing.

### 5.5 Immunofluorescence and Immunohistochemistry

Immunofluorescence (IF) and immunohistochemistry (IHC) were performed on human surgical specimens and mouse tumor tissues using standardized protocols. FFPE blocks were cut at 4 µm, deparaffinized, rehydrated, and subjected to heat‐mediated antigen retrieval (citrate buffer pH 6.0 or Tris‐EDTA pH 9.0 as indicated). For IHC, endogenous peroxide was quenched, and sections were blocked with 5% normal serum. Primary antibodies were applied overnight at 4°C. Signal was developed with HRP‐conjugated secondary antibodies and DAB chromogen, and sections were counterstained with hematoxylin. H‐scores were assigned by a pathologist blinded to treatment groups. For IF, frozen sections or antigen‐retrieved FFPE sections were permeabilized with 0.1% Triton X‐100 when intracellular targets were probed, then blocked with 3% BSA or normal serum. Primary antibodies were followed by species‐matched fluorescent secondary antibodies. CAR localization on Jurkat cells was detected using anti‐tag reagents on cytospins sections. Images were acquired on fluorescence microscopes with identical exposure settings across comparative samples and were processed in ImageJ. Quantification was performed on ≥5 non‐overlapping fields per sample using uniform thresholds, and all image capture and analysis were conducted with the operator blinded to experimental group. Negative controls (isotype or no‐primary antibody) were included in every staining run.

### 5.6 Flow Cytometry

Single‐cell suspensions from cell lines, primary T cell products, spleen, or tumor digests were stained and analyzed by flow cytometry to assess lineage, activation, exhaustion, differentiation, and cell‐cycle status. Cells were washed in PBS and resuspended in FACS buffer (PBS with 2% FBS). Live/dead discrimination was performed using a fixable viability dye according to the manufacturer's instructions. Fc receptors were blocked where appropriate. Surface staining was performed with fluorophore‐conjugated antibodies or with unconjugated primary antibodies followed by species‐matched fluorescent secondary antibodies. Staining was carried out at 4°C for 20–30 min in the dark. For intracellular targets, cells were fixed and permeabilized using a commercial fixation/permeabilization kit and stained with the appropriate antibody following the supplier protocol. Cell‐cycle analysis was performed by ethanol fixation followed by RNase A treatment and propidium iodide staining. Single‐color compensation controls and fluorescence minus one (FMO) controls were included for gating. Data were acquired on a BD cytometer and analyzed in FlowJo. Statistical analyses were carried out in R (R Studio).

### 5.7 Western Blotting

Cells were lysed in RIPA buffer supplemented with protease and phosphatase inhibitors and cleared by centrifugation. Protein concentration was measured by BCA, and 20–40 µg protein per lane was separated by SDS‐PAGE and transferred to PVDF membranes. For phospho‐detection, membranes were blocked in 5% BSA, otherwise 5% milk was used. Membranes were incubated with primary antibodies overnight at 4°C, washed, incubated with HRP‐conjugated secondary antibodies, developed with ECL reagent, and imaged on a chemiluminescence system. Bands were quantified using ImageJ and normalized to total protein or loading control. All blots were performed in ≥3 independent biological replicates, and antibody details are provided in the Key Resources Table.

### 5.8 CRISPR/Cas9 Knockout and Rescue

Two sgRNAs targeting exon 3 of CSPG4 were designed and cloned into a CRISPR‐Cas9 backbone (Table S2) [79]. HNSCC cells were transfected or transduced with CRISPR constructs and selected as appropriate to generate polyclonal knockout populations; individual clones were isolated when required. Knockout was validated by Western blot and flow cytometry. For rescue, a CSPG4 cDNA resistant to sgRNA targeting was cloned into an expression vector and re‐introduced by lentiviral transduction [80]. Re‐expression was confirmed by Western blot and flow cytometry.

### 5.9 shRNA Lentivirus Production and Transduction

To generate shRNAs targeting the human CD44 gene, the corresponding shRNA oligonucleotides were cloned into the pLKO.1 vector via AgeI and EcoRI restriction sites (see Table S2). Following sequence confirmation, the shRNA constructs were packaged into lentiviral particles through co‐transfection of HEK293T cells with the packaging plasmids pMD2.G and psPAX2. The produced lentiviruses encoding CD44 shRNA were then used to transduce the specified cell lines. Stably transduced cells were subsequently selected under 1 µg/ml puromycin treatment for three days.

### 5.10 Proliferation Assay

Proliferation of HNSCC cell lines was assessed using the Cell Counting Kit‐8. Cells were seeded into 96‐well plates at the indicated densities and cultured for the specified time points. At each time point, CCK‐8 reagent was added to each well and incubated at 37°C for 1–2 h. Absorbance at 450 nm was measured using a microplate reader, and cell proliferation curves were generated based on optical density values.

### 5.11 Cell Viability Assays

For the cell viability assay, tumor cells were plated in 96‐well flat‐bottom plates at a seeding density of 5000 cells per well and allowed to attach overnight. The cells were then treated with a range of cisplatin concentrations for 24 h. Following treatment, cell viability was assessed using the CCK‐8 assay.

### 5.12 Colony Formation Assay

Tumor cells were suspended in complete RPMI‐1640 medium containing 10% fetal bovine serum (FBS) and seeded into 12‐well plates at a density of 500 cells per well. After 12 days of culture under standard conditions, the cells were fixed with 4% paraformaldehyde at room temperature for 20 min and subsequently stained with 0.1% crystal violet for 15 min. Following the removal of unbound stain, colonies were visualized under a dissection microscope. The number of colonies consisting of more than 50 cells was counted, and the results were used to assess colony formation efficiency.

### 5.13 Tumor Sphere Formation Assay

For the tumor sphere formation assay, tumor cells were plated at a density of 1000 cells per well into ultra‐low attachment plates (LABSELECT, Cat. No. 11118). The cells were cultured in serum‐free DMEM/F12 medium (meilunbio, Cat. No. MA0214‐2) supplemented with 1×B27 (Thermo Fisher Scientific, Cat. No. 41400045), 20 ng/mL human recombinant epidermal growth factor (EGF; novoprotein, Cat. No. C029), and 10 ng/mL human recombinant basic fibroblast growth factor (bFGF; novoprotein, Cat. No. C046). Incubation was carried out at 37°C in a humidified 5% CO_2_ atmosphere. After seven days, spheres were collected, and those exceeding 50 µm in diameter were enumerated.

### 5.14 Wound‐Healing Assay

Cells were seeded in 6‐well plates and grown to near confluence (≥ 90%) in complete medium at 37°C with 5% CO_2_. A linear wound was created in each monolayer using a sterile 200‐µL pipette tip, and detached cells were removed by gently washing twice with PBS. Cells were then incubated in medium containing low serum (1% FBS) to minimize proliferation. Images of the same wound field were acquired immediately (0 h) and at defined intervals thereafter (typically 24 and 48 h) using a phase‐contrast microscope. Wound areas were measured from micrographs using ImageJ.

### 5.15 Transwell Invasion Assay

Cell invasion was measured using 24‐well Transwell inserts with 8 µm pores (Corning) precoated with growth factor‐reduced Matrigel (BD Biosciences). Inserts were coated with 50 µL Matrigel diluted 1:8 in serum‐free medium and allowed to solidify at 37°C for 1 h. Cells were serum‐starved for 6–12 h, trypsinized, and resuspended in serum‐free medium. Between 1 × 10^4^ and 5 × 10^4^ cells in 200 µL were seeded into the upper chamber. The lower chamber was filled with 600 µL complete medium containing 10% FBS as a chemoattractant. Plates were incubated at 37°C in 5% CO_2_ for 16–24 h depending on cell line. After incubation non‐invading cells and Matrigel were removed from the upper surface with a cotton swab. Invaded cells on the lower membrane surface were fixed with 4% paraformaldehyde for 10 min, stained with 0.1% crystal violet for 10–15 min, rinsed gently, and air‐dried. Membranes were imaged under a light microscope, and invaded cells were counted in at least five random fields per insert at 10× or 20× magnification.

### 5.16 RNA Sequencing

Total RNA was extracted from tumor or CAR‐T cells and prepared for sequencing using polyA selection or a modified SMART‐Seq2 protocol, respectively [76]. All libraries were sequenced on an Illumina NovaSeq system, with raw reads aligned to the GRCh37/hg19 reference genome using Samtools v1.18 and quantified via featureCounts v2.0.6. Differential expression analysis was performed with DESeq2 (adjusted *p* < 0.05), followed by comprehensive functional characterization using clusterProfiler for GO enrichment [77], the GSVA R package for Hallmark and ssGSEA against Molecular Signatures Database gene set (https://www.gsea‐msigdb.org/gsea/msigdb), and GeneMANIA (https://genemania.org/search/homo‐sapiens) for network modeling. All visualizations, including heatmaps and volcano plots, were generated using custom R scripts (pheatmap and ggplot2). The raw RNA‐seq data generated in this study have been deposited in the Gene Expression Omnibus (GEO) database under the accession number GSE315646.

### 5.17 Lentiviral CAR Constructs and Production

The amino acid sequences for the variable heavy (VH) and variable light (VL) chains of the murine monoclonal antibody 763.74 were obtained from the seminal publication [26]. After being codon‐optimized, VH and VL sequences were linked by a standard G4S linker to form the scFv. This DNA fragment encoding the 763.74 scFv was commercially synthesized de novo. For clarity, the recombinant scFv derived from the 763.74 sequence was used exclusively as the antigen‐binding domain within the CAR construct. CAR constructs were cloned into the phR lentiviral backbone according to the designs described in the manuscript. Lentiviral particles were produced by transient transfection of HEK293T cells using a three‐plasmid system (transfer plasmid, psPAX2 packaging plasmid, pMD2.G envelope plasmid) and concentrated by ultracentrifugation or PEG precipitation as needed. Viral titers were estimated by transduction of permissive cells.

### 5.18 CAR Humanization and Charge‐Optimization

The humanized CSPG4‐specific scFv was designed through a multi‐step rational process to minimize immunogenicity and reduce tonic signaling (summarized in Figure S10). First, the CDRs of the murine 763.74 VH and VL chains were precisely defined using the IMGT/V‐QUEST tool (www.imgt.org/IMGT_vquest) to ensure accurate boundary annotation according to the IMGT numbering scheme. Human germline framework regions (FRs) were then retrieved from the IMGT/GENE‐DB database (www.imgt.org/genedb) [81]. Candidate human FRs were systematically screened, and selection was based primarily on the lowest cumulative frequency of positively charged residues (Lysine, Arginine, and Histidine) within their sequences to inherently minimize surface positive charge. The murine CDRs were subsequently grafted onto the selected human FRs to generate the humanized VH and VL sequences.

The humanized variable regions underwent a two‐step in silico validation process. For humanness assessment, the amino acid sequences of the humanized VH and VL were submitted separately to the online tool http://www.bioinf.org.uk/abs/shab/. This server compares query sequences against a curated database of human and murine antibody sequences and returns a humanization (H‐) score for each chain [82]. Analysis confirmed a significant increase in H‐scores for both chains, placing them well within the range of natural human antibody sequences.

For surface charge validation, the humanized VH and VL were connected with a G4S linker to form the final scFv. Its 3D structure was predicted by homology modeling using the SWISS‐MODEL server [82]. The electrostatic potential characteristics were analyzed using Swiss‐PdbViewer software (version 4.1.0). [83] Electrostatic surfaces were then computed with the APBS solver integrated into UCSF Chimera (version 1.14) for visualization [84]. Finally, the BindUp web service was employed to identify and quantify positive‐charge clusters, calculating the PCP score as the summed surface area of the three largest positive patches [85]. This comprehensive analysis demonstrated successful charge optimization of the humanized scFv construct.

### 5.19 Primary Human T Cell Isolation, Transduction and Expansion

Peripheral blood mononuclear cells (PBMCs) were obtained from healthy donors. CD3^+^ T cells were isolated by negative selection and activated with anti‐CD3/CD28 beads in X‐VIVO 15 medium supplemented with IL‐2. Activated T cells were transduced with concentrated lentivirus (spinoculation) at MOIs optimized to achieve the stated CAR positivity (30%–60%), then expanded for 7–14 days. CAR expression and T cell phenotypes were assessed by flow cytometry.

### 5.20 Cytotoxicity Assay

Tumor cells stably expressing firefly luciferase were seeded into 96‐well white plates and co‐cultured with CAR‐T or non‐transduced T cells at the indicated effector‐to‐target (E:T) ratios in triplicate. After 24 h at 37°C, D‐luciferin (150 µg/mL) was added, and luminescence was measured using a microplate reader. Percent specific lysis was calculated as 100×[1−(RLU_sample_/RLU_target only_)].

### 5.21 Elisa

Cytokine secretion by CAR‐T cells was quantified using an enzyme‐linked immunosorbent assay (ELISA). Briefly, CAR‐T cells were co‐cultured with target tumor cells at the indicated effector‐to‐target (E:T) ratios in complete RPMI 1640 medium in 96‐well plates. After 24 h of incubation at 37°C with 5% CO_2_, supernatants were collected and clarified by centrifugation at 300× g for 5 min. Concentrations of human IFN‐γ, TNF‐α, and IL‐2 were measured using commercial ELISA kits according to the manufacturer's instructions. Optical density was read at 450 nm using a microplate reader with wavelength correction at 570 nm. Cytokine concentrations were calculated from standard curves generated with recombinant cytokines provided in the kits. All assays were performed in duplicate or triplicate and repeated in at least three independent experiments.

### 5.22 In Vivo Xenograft, Metastasis and PDX Studies

Immunocompromised NOD‐SCID‐Il2rg −/− (NSG) mice were obtained from Biocytogen. All mice were bred and maintained under specific pathogen‐free conditions at the National Facility for Protein Science in Shanghai, in compliance with institutional ethical guidelines (approval number: 20250110002). For subcutaneous xenografts, HNSCC cell lines (wild‐type, CSPG4‐KO, or Rescue) were implanted into the flanks of NSG mice. Tumor volumes were measured periodically by calipers or monitored by bioluminescence imaging when tumor cells expressed luciferase. For the experimental metastasis model, cells were injected via tail vein, and mice monitored for survival. PDX models were established from CSPG4‐positive patient tumors and expanded in NSG mice; PDX‐bearing mice received intravenous infusion of control, CSPG4^WT^.CAR‐T, or CSPG4^Hu^.CAR‐T cells. Body weight and clinical signs were monitored; tumor burden was quantified by caliper measurement or bioluminescence, and survival was analyzed by the Kaplan–Meier method. At endpoints, spleens and tumors were harvested for flow cytometry, immunofluorescence, and histology. Exact cell numbers, CAR‐T dosing, and time points are provided in figure legends and in Supplementary Methods.

### 5.23 Hematoxylin and Eosin (H&E) Staining

Formalin‐fixed paraffin‐embedded sections were cut at 4 µm, deparaffinized in xylene, and rehydrated through graded ethanol to water. Sections were stained in Harris hematoxylin for 3–5 min, rinsed in running tap water, differentiated in 1% acid alcohol if required, and blued in Scott's tap water substitute or 0.2% ammonia water. Slides were then counterstained with eosin for 30–60 s, dehydrated through ascending ethanols, cleared in xylene, and coverslipped with a permanent mounting medium. Stained sections were reviewed and photographed under brightfield microscopy and assessed by a pathologist blinded to experimental groups.

### 5.24 Quantification and Statistical Analysis

All statistical analyses were performed using R (R Studio) unless otherwise indicated. Flow cytometry data were analyzed with FlowJo. Data are presented as mean ± SEM unless stated otherwise. For comparisons between two groups, a two‐tailed Student's *t*‐test was used for normally distributed data; for multiple groups, one‐way ANOVA with appropriate post‐hoc tests (Tukey or Dunnett) was applied. Survival analyses used Kaplan–Meier estimation and the log‐rank test. Differential expression analyses for RNA‐seq employed DESeq2 with Benjamini–Hochberg correction for multiple testing; adjusted *p*‐values < 0.05 were considered significant. CIBERSORT deconvolution used 1000 permutations and the standard LM22 signature. TIDE scores were compared using Wilcoxon rank‐sum tests. Exact n (biological replicates) and statistical tests are specified in figure legends.

## Funding

National Natural Science Foundation of China (82471150 and 82102863 to J.C., 82473310 and 82271149 to H.W., and 81870710 to P.H.), the Shanghai Rising‐Star Program (23QA1400900 to J.C.), the Youth Medical Talents – Specialist Program (SHWSRS (2023) _062 to J.C.), and the Shanghai Clinical Research Center for Cell Therapy (23J41900100).

## Conflicts of Interest

J.C., H.W., S.Q., M.C., and Y.F. has filed and obtained authorization for a Chinese patent related to the humanized CSPG4.CAR sequence (Patent No.: ZL202010477197.3).

## Supporting information




**Supporting File**: advs74442‐sup‐0001‐SuppMat.docx

## Data Availability

The data that support the findings of this study are available from the lead corresponding author (chenj15@fudan.edu.cn) upon reasonable request.
