# Supplementary material for: Humanized and Charge‐Optimized CSPG4‐Specific CAR‐T Cells show Enhanced Efficacy against Head and Neck Squamous Cell Carcinoma
Source: Adv Sci (Weinh). 2026 Feb 16;13(35):e19746. doi: 10.1002/advs.202519746 (PMC13292224; doi:10.1002/advs.202519746)
Supplement: Supplementary file 1 — Supporting File: advs74442‐sup‐0001‐SuppMat.docx [file ADVS-13-e19746-s001.docx]

Supporting Information

Humanized and Charge-Optimized CSPG4-Specific CAR-T Cells Show Enhanced Efficacy Against Head and Neck Squamous Cell Carcinoma

*Xiang Xu, Shizhen Qiu, Zhitong Wang, Dan Li, Min Chen, Yinying Chu, Guangfei Li,1 Yi Fang, Changjiang Li, Fang Shi, Peijie He,* Haitao Wu,* Haopeng Wang,* and Jian Chen,**


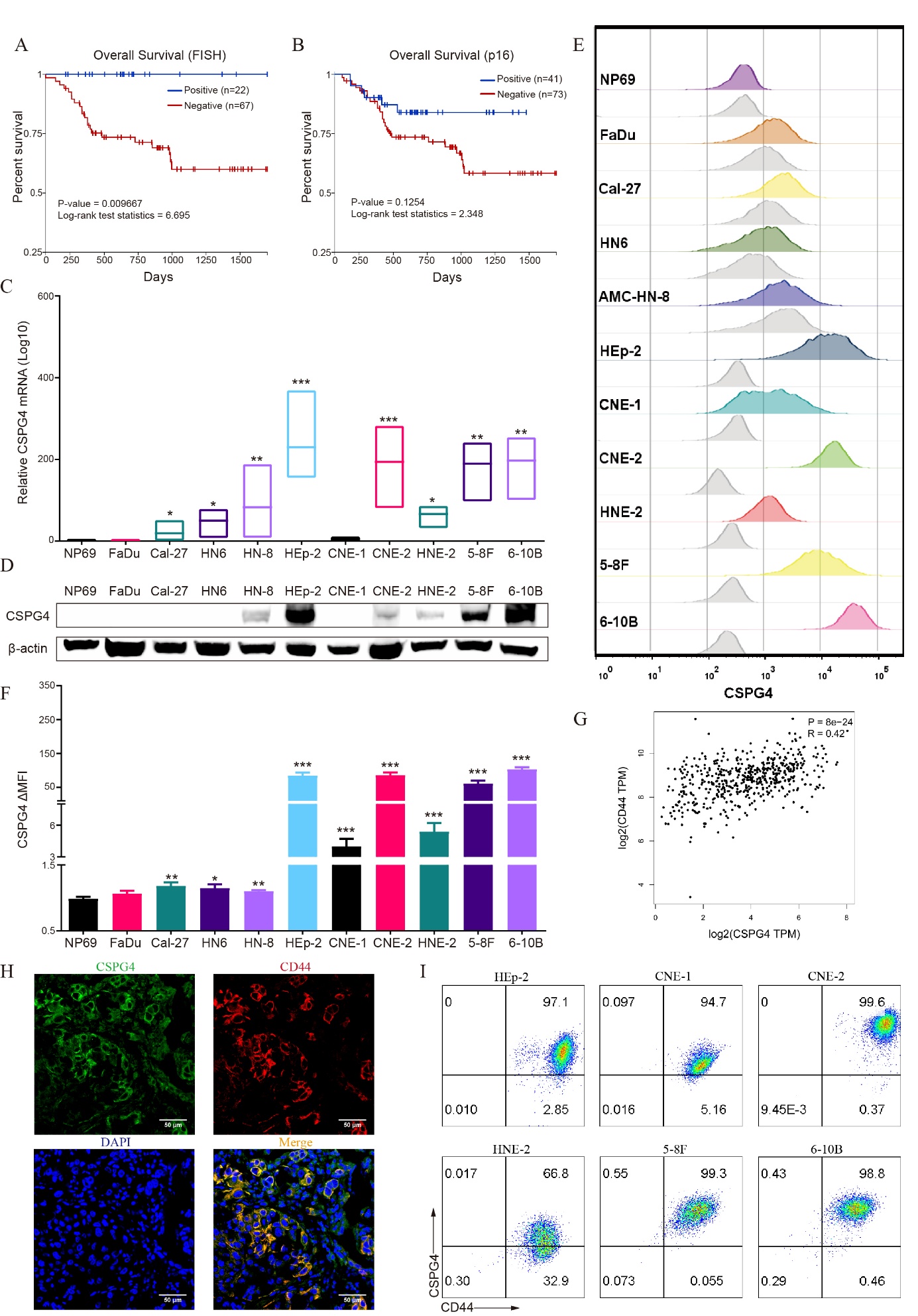


**Figure S1.** **CSPG4 expression profiles across HPV status and its correlation with CD44 in HNSCC.**

(A) Kaplan–Meier survival analysis of TCGA HNSCC patients stratified by HPV status determined by FISH. (B) Kaplan–Meier survival analysis of TCGA HNSCC patients stratified by HPV status determined by p16 staining. (C) qPCR quantification of CSPG4 mRNA in ten HNSCC cell lines (FaDu, Cal-27, HN6, HN-8, HEp-2, CNE-1, CNE-2, HNE-2, 5-8F, 6-10B) relative to the normal nasopharyngeal epithelial line NP69. (D) Western blot analysis of CSPG4 protein in the same panel of HNSCC cell lines, with NP69 as a negative control. (E) Representative flow cytometry histograms showing surface CSPG4 expression on HNSCC cell lines and NP69 control. (F) Quantification of CSPG4 surface expression from panel E. (G) Correlation analysis of CSPG4 and CD44 mRNA expression in TCGA HNSCC samples. (H) Representative immunofluorescence for CSPG4 (green), CD44 (red), and DAPI (blue) in patient HNSCC tissue sections (Scale bar = 50 μm). (I) Flow cytometry dot plots profiling the surface co-expression of CSPG4 and CD44 on representative HNSCC cell lines. Error bars represent Mean ± SEM. Statistical significance was determined by Student’s t-test or one-way ANOVA (*p < 0.05, **p < 0.01, ***p < 0.001).


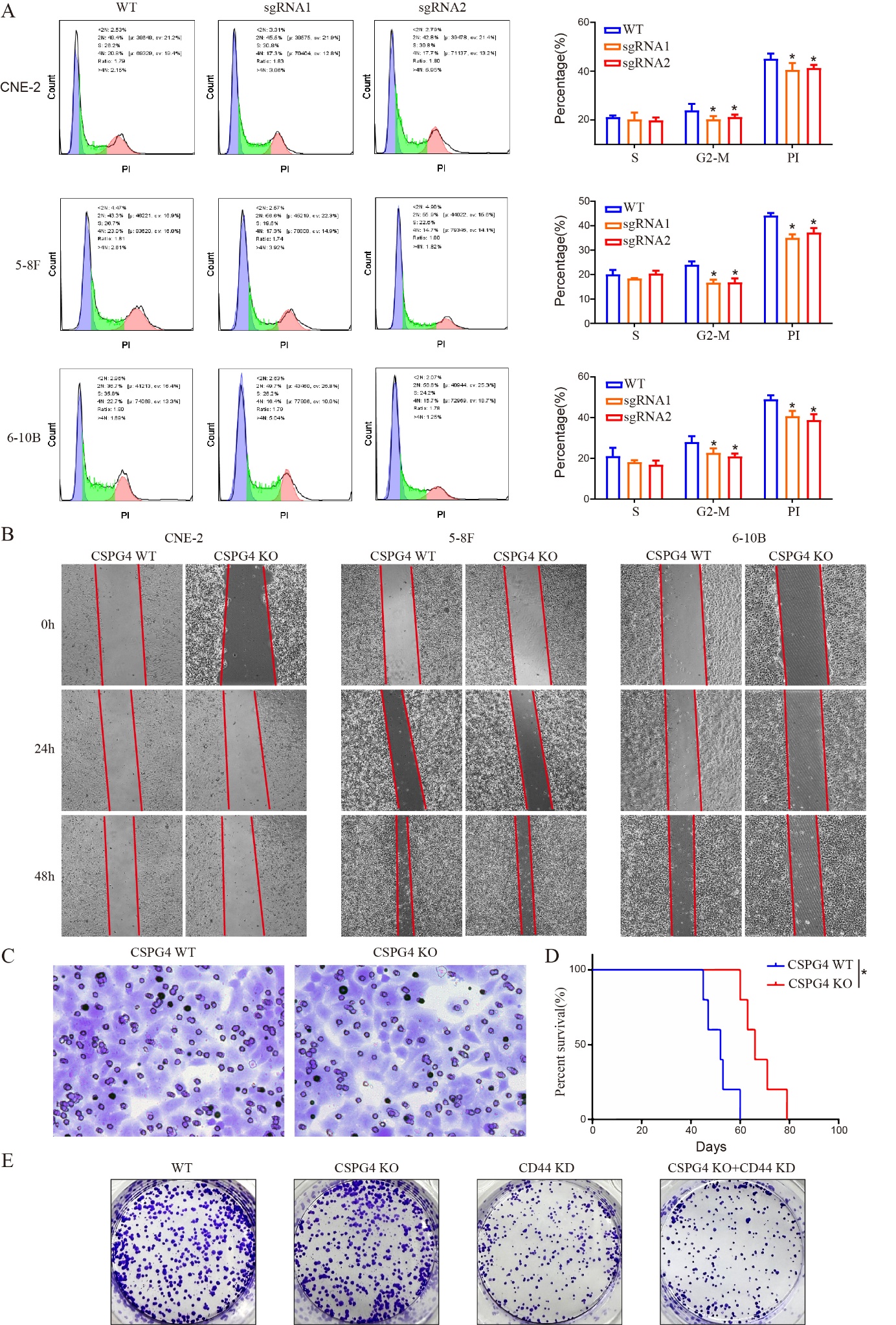


**Figure S2. Impact of CSPG4 knockout on cell cycle, migration, invasion, and systemic metastasis.**

A) Flow cytometric cell-cycle profiling and calculated proliferation index in CSPG4-KO versus wild-type lines. B) Representative images and quantification of the scratch-wound healing assay in HNSCC cells. C) Transwell invasion assay through Matrigel-coated membranes for CSPG4-KO and wild-type cells. D) Kaplan–Meier survival analysis of NSG mice following systemic tail-vein injection of CNE-2 cells. E) Representative results and statistical quantification of colony formation in the indicated isogenic lines. Error bars represent Mean ± SEM (*p < 0.05).


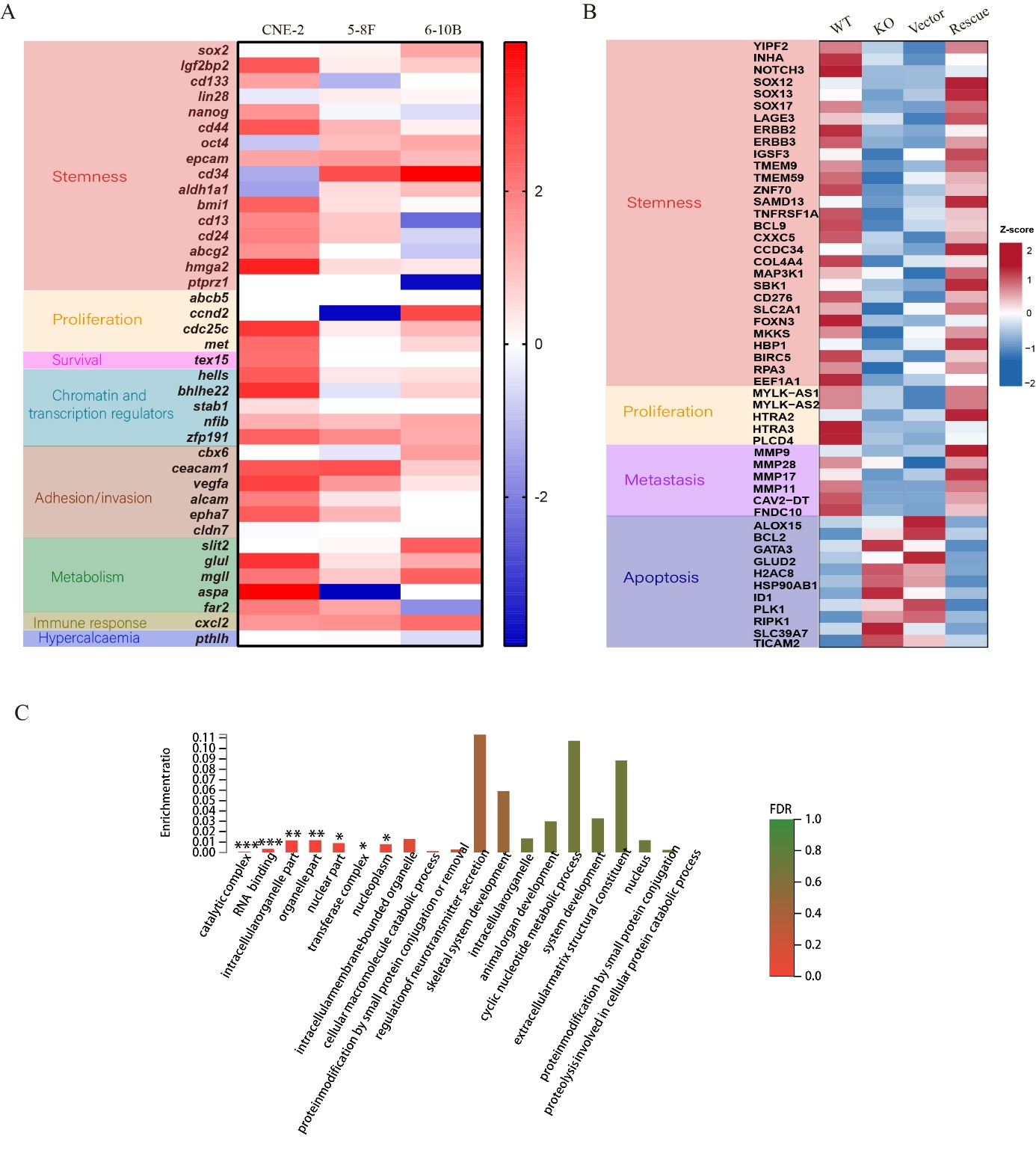


**Figure S3. Transcriptomic changes following CSPG4 knockout and rescue in HNSCC cell lines.**

(A) qPCR-based heatmap of transcripts involved in oncogenic pathways following CSPG4 knockout in three HNSCC cell lines. (B) RNA-seq heatmap showing the expression of stemness, proliferation, metastasis, and apoptosis genes in WT, KO, Vector, and Rescue CNE-2 cells. (C) GO enrichment analysis of differentially expressed genes in CSPG4-KO CNE-2 cells. Error bars represent Mean ± SEM (*p < 0.05, **p < 0.01, ***p < 0.001).


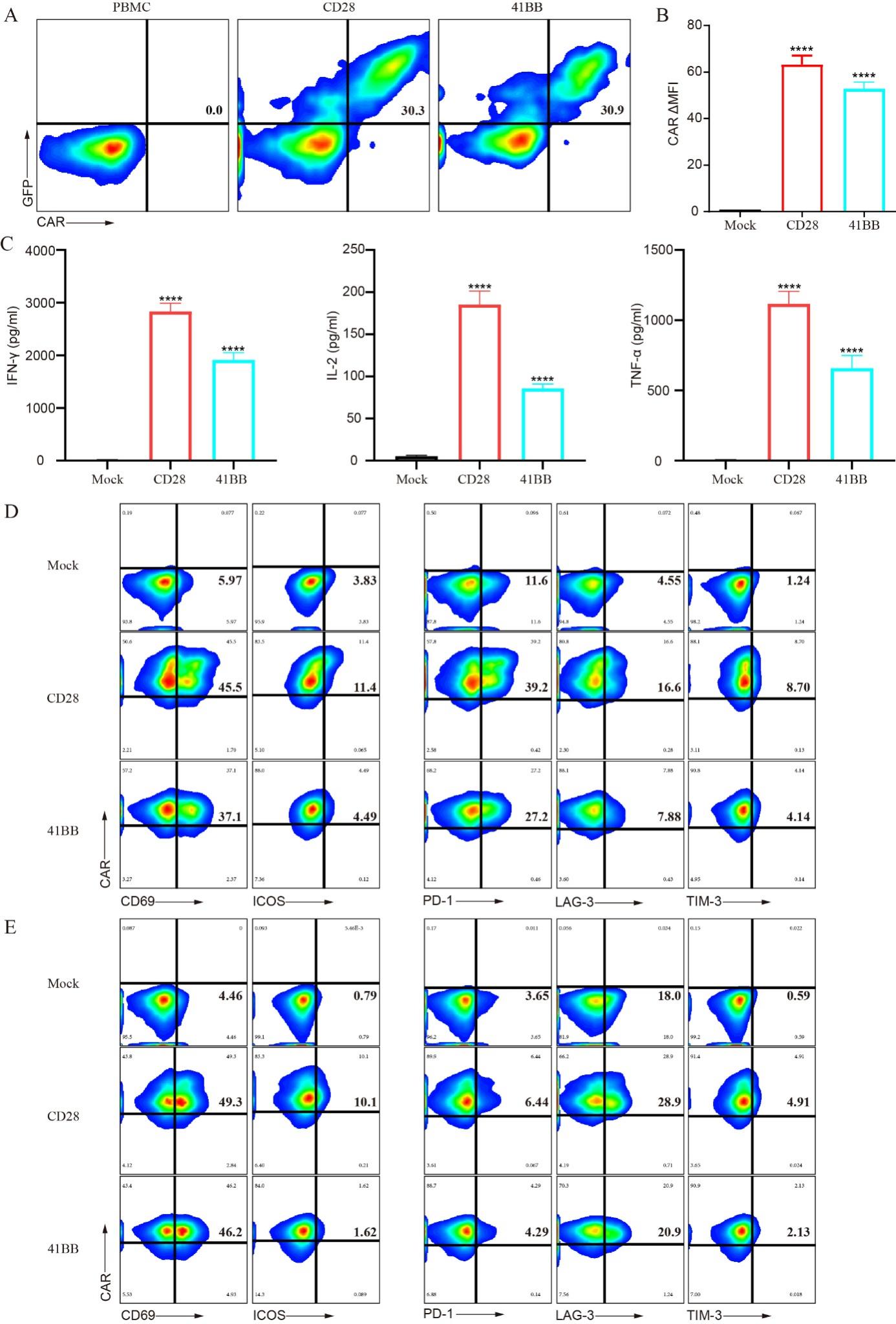


**Figure S4.** **Assessment of tonic signaling and basal exhaustion in CSPG4.CAR-T cells.**

A-B) Representative flow cytometry plots (A) and statistical quantification (B) of CAR surface expression on mIgG4-hinge CAR-T cells. C) ELISA-based quantification of IFN-γ, IL-2, and TNF-α secretion by CAR-T cells in the resting state. D-E) Flow cytometric analysis of activation (CD69, ICOS) and exhaustion (PD-1, LAG-3, TIM-3) markers on resting CD4^+^ (D) and CD8^+^ (E) CAR-T cell subsets. Error bars represent Mean ± SEM (****p < 0.0001).


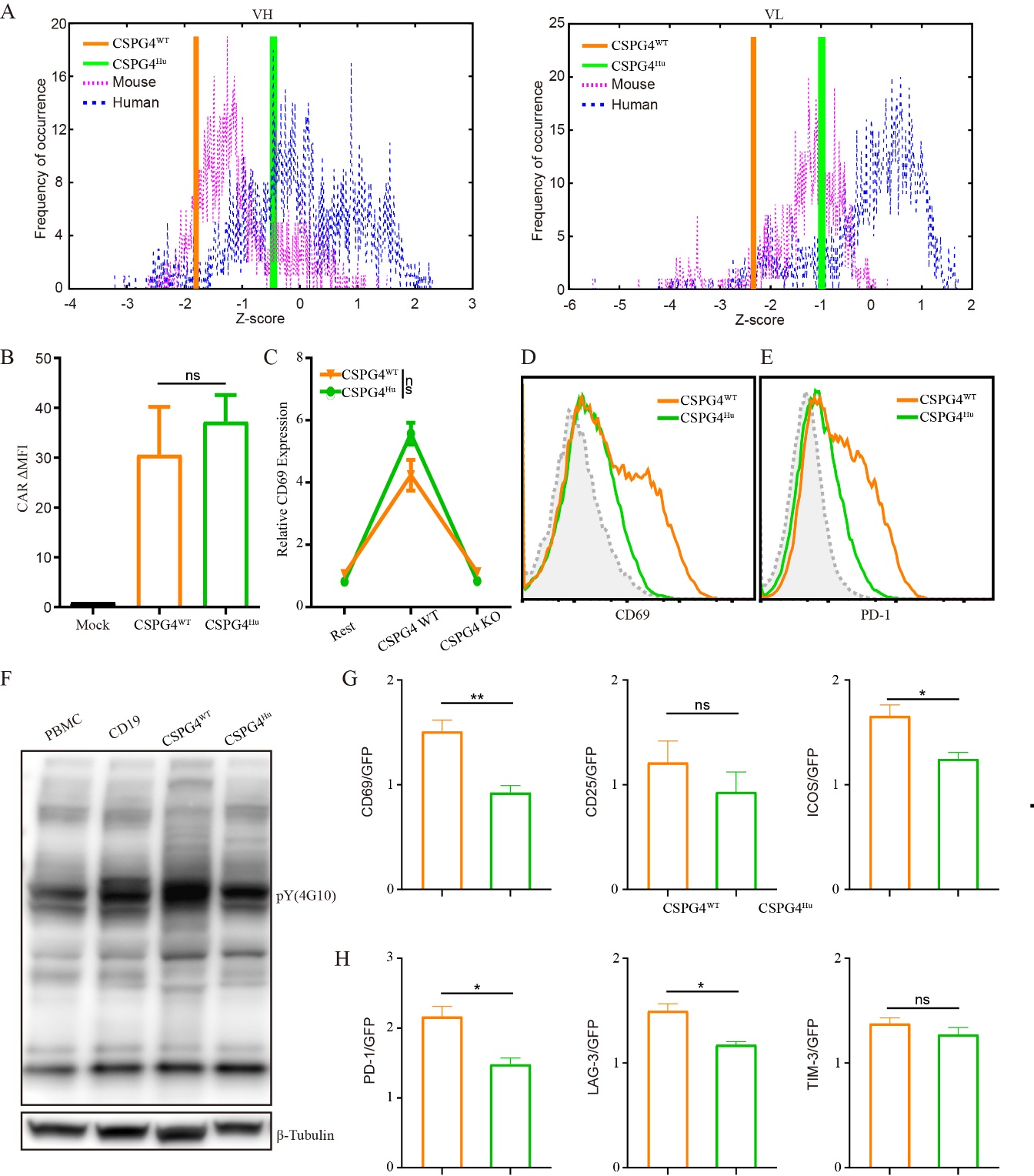


**Figure S5. Biophysical and signaling characterization of the humanized CSPG4.CAR.**

A) Humanness distribution for the optimized heavy and light chains. B-C) Surface CAR expression (B) and antigen-specific CD69 upregulation (C) in Jurkat-CAR cells. D-E) Representative histograms of basal CD69 (D) and PD-1 (E) expression in resting Jurkat-CAR cells. F) Western blot of total phosphotyrosine (pY) levels in resting CAR-T cells. G-H) Quantification of surface activation markers (CD69, CD25, ICOS) (G) and exhaustion markers (PD-1, LAG-3, TIM-3) (H) in resting primary CAR-T cells.


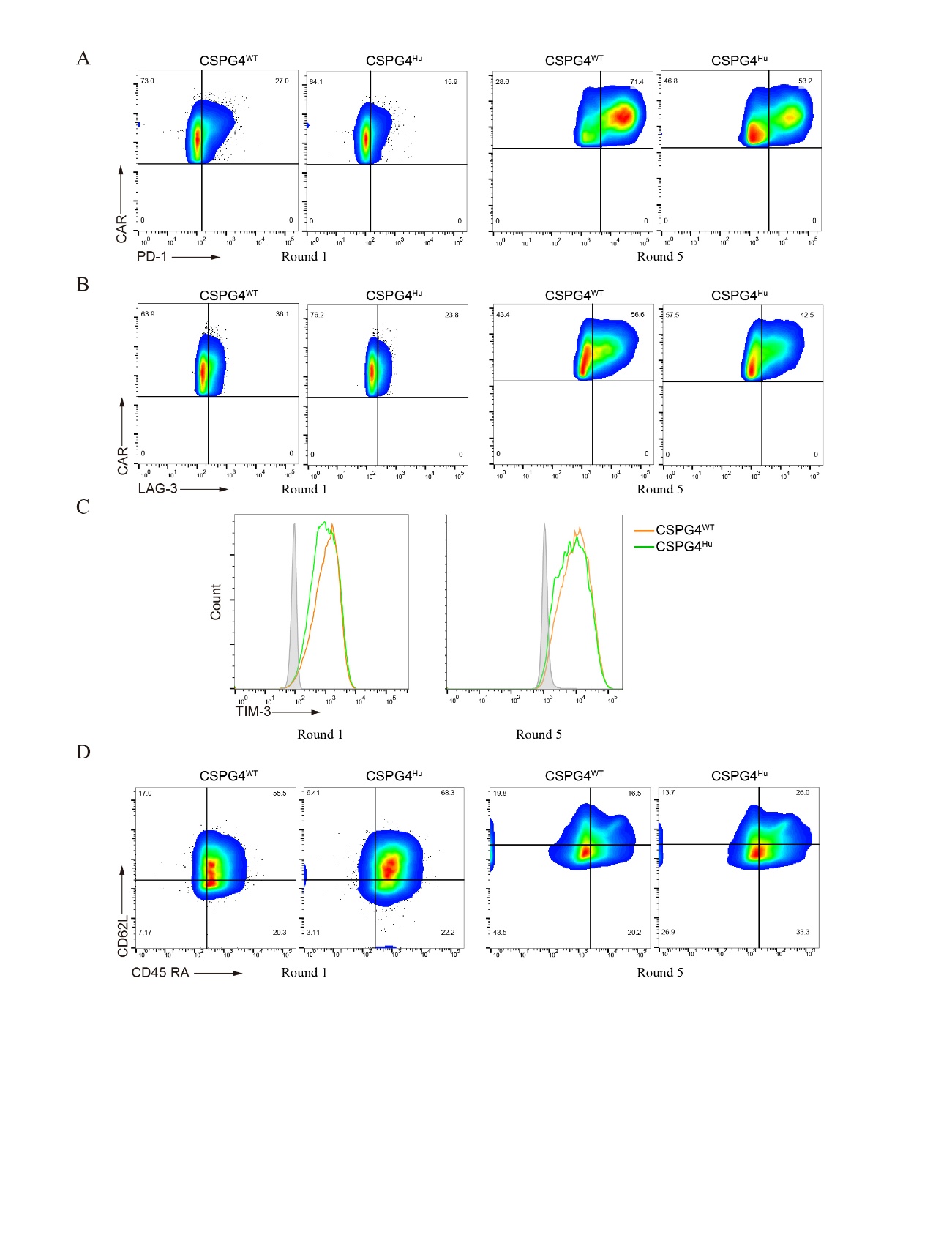


**Figure S6. Flow cytometric profiling of CAR-T cells during serial re-stimulation.**

A–B) Representative flow cytometry plots showing PD-1 (A) and LAG-3 (B) expression on CAR-T cells after Round 1 and Round 5 of co-culture. C) Histograms showing TIM-3 expression levels following serial challenge. D) Representative flow plots of the Tscm​ (CD45RA^+^ CD62L^+^) population, demonstrating the superior memory retention of CSPG4^Hu^.CAR-T cells after five rounds of stimulation.


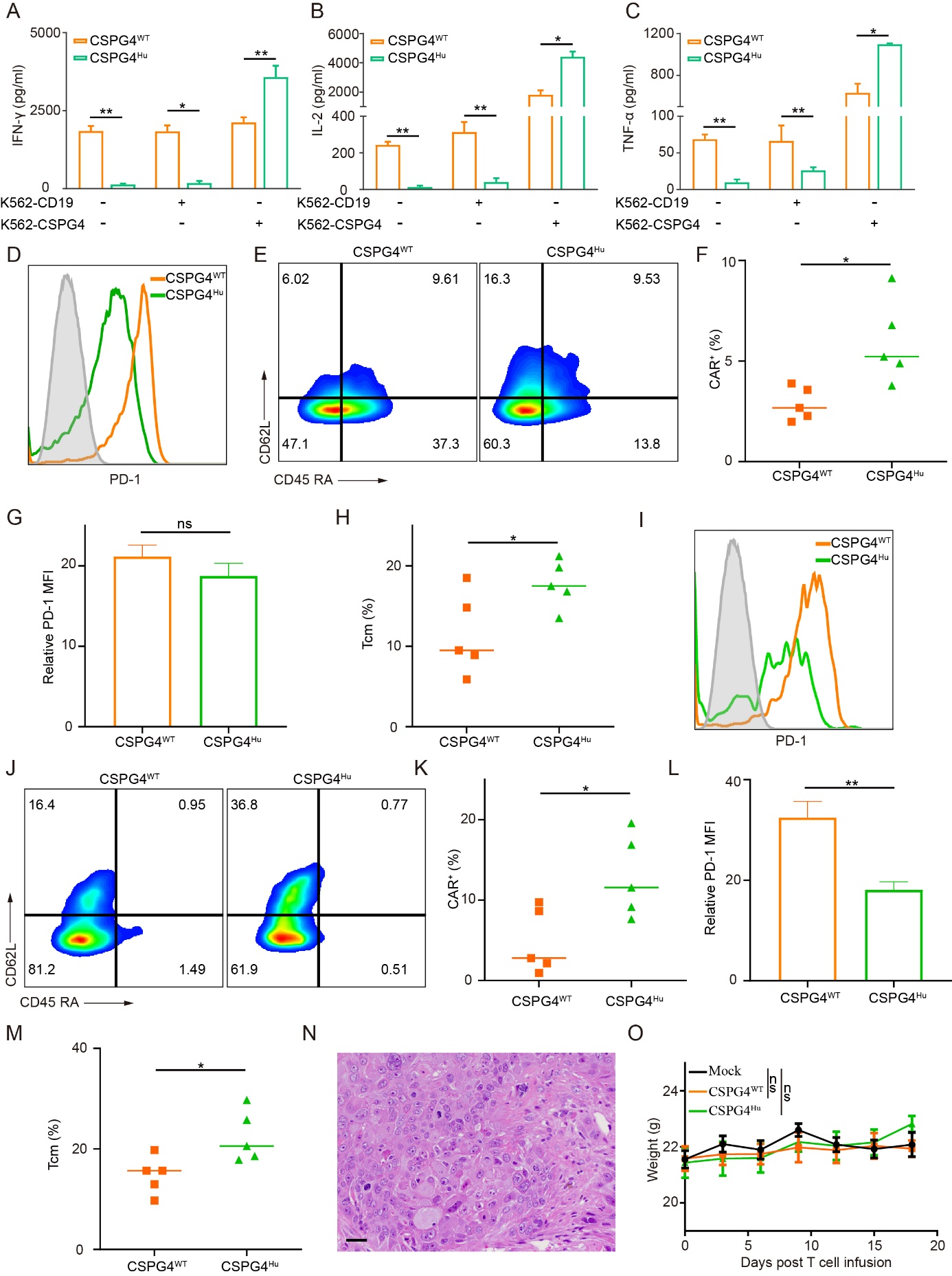
**Figure S7. Phenotypic profiling of CAR-T cells in the blood and TME.**

A-I) Analysis of CD4+ CAR-T cells in the blood, including frequency, PD-1 levels, and memory subsets. J-M) Analysis of CD4^+^ TILs within the tumor. N) H&E staining of the HNSCC PDX tumor tissue (Scale bar = 50 μm). O) Body weight monitoring of mice during the treatment period.


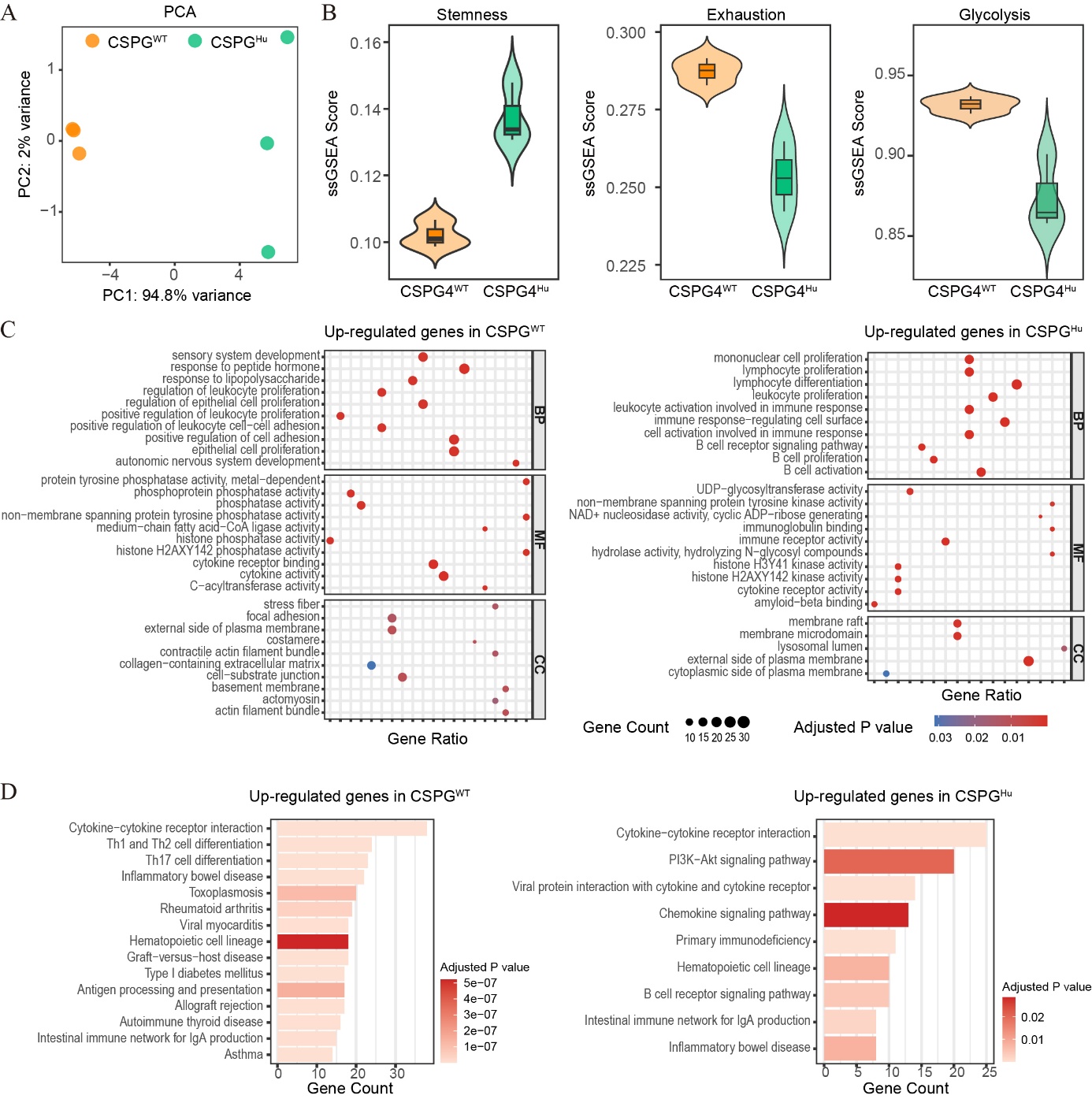


**Figure S8. Mechanistic insights into the enhanced fitness of CSPG4hu CAR-T cells via transcriptomic analysis.**

A) Principal Component Analysis (PCA) showing distinct transcriptomic separation between CSPG4^WT^ (orange) and CSPG4^Hu^ (green) CAR-T cells. B) ssGSEA scores for stemness, exhaustion, and glycolysis. C) GO enrichment analysis of upregulated genes in CSPG4^WT^ (Left) and CSPG4^Hu^ (Right) CAR-T cells. D) KEGG pathway enrichment analysis of upregulated genes in CSPG4^WT^ (Left) and CSPG4^Hu^ (Right) CAR-T cells. ssGSEA, single-sample Gene Set Enrichment Analysis.


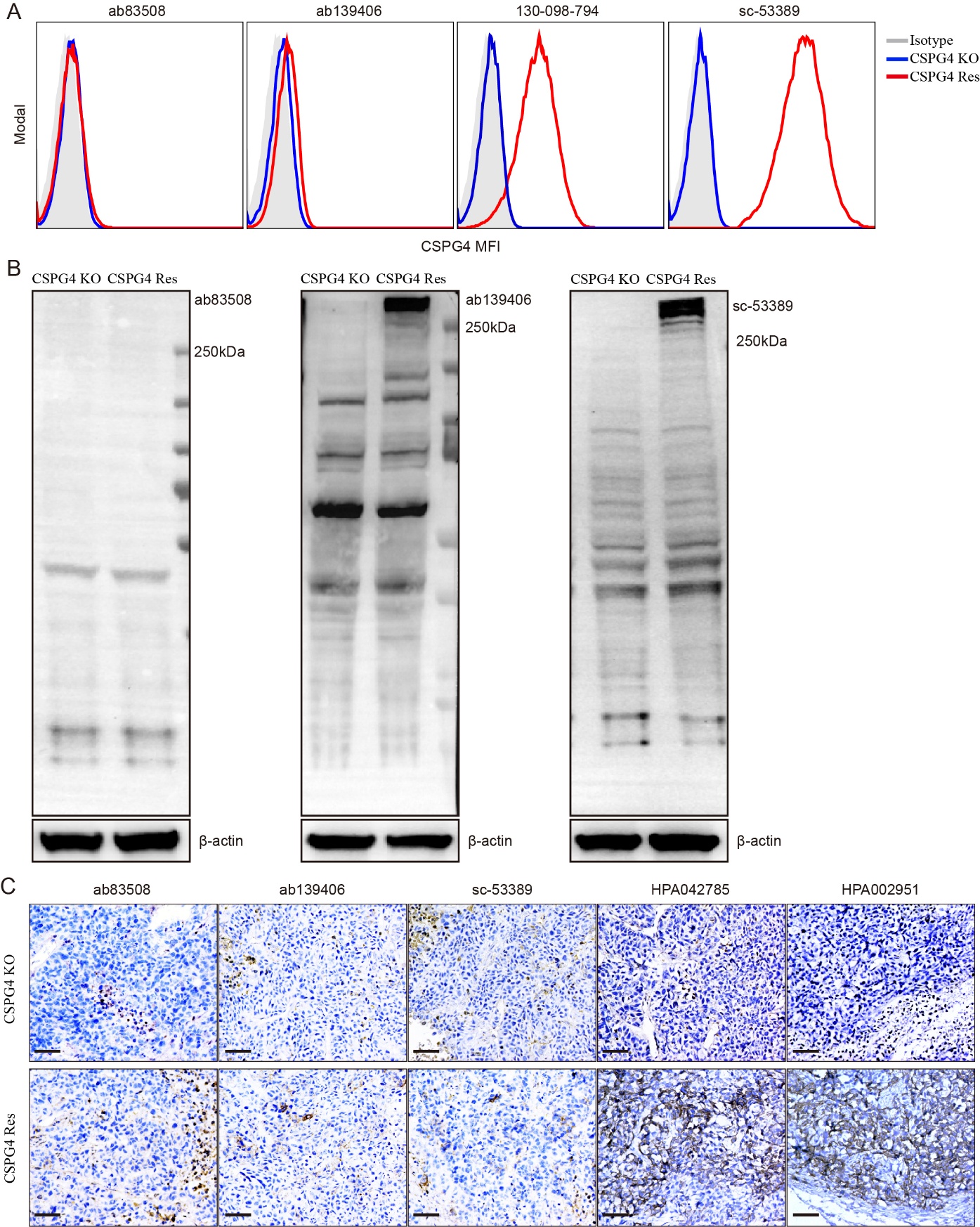


**Figure S9. Systematic screening and validation of platform-specific anti-CSPG4 antibodies.**

A) Flow Cytometry (FACS) Antibody Screening. Representative flow cytometry histograms showing the cell surface staining of various anti-CSPG4 clones. B) Western Blot Antibody Validation. Western blot analysis of CSPG4 core protein expression in CSPG4-KO and CSPG4-Rescue cell lysates. C) Immunohistochemistry Antibody Validation. Representative IHC images of sections from subcutaneous tumor xenografts derived from CSPG4-KO and CSPG4-Rescue cells (Scale bar = 100 μm).


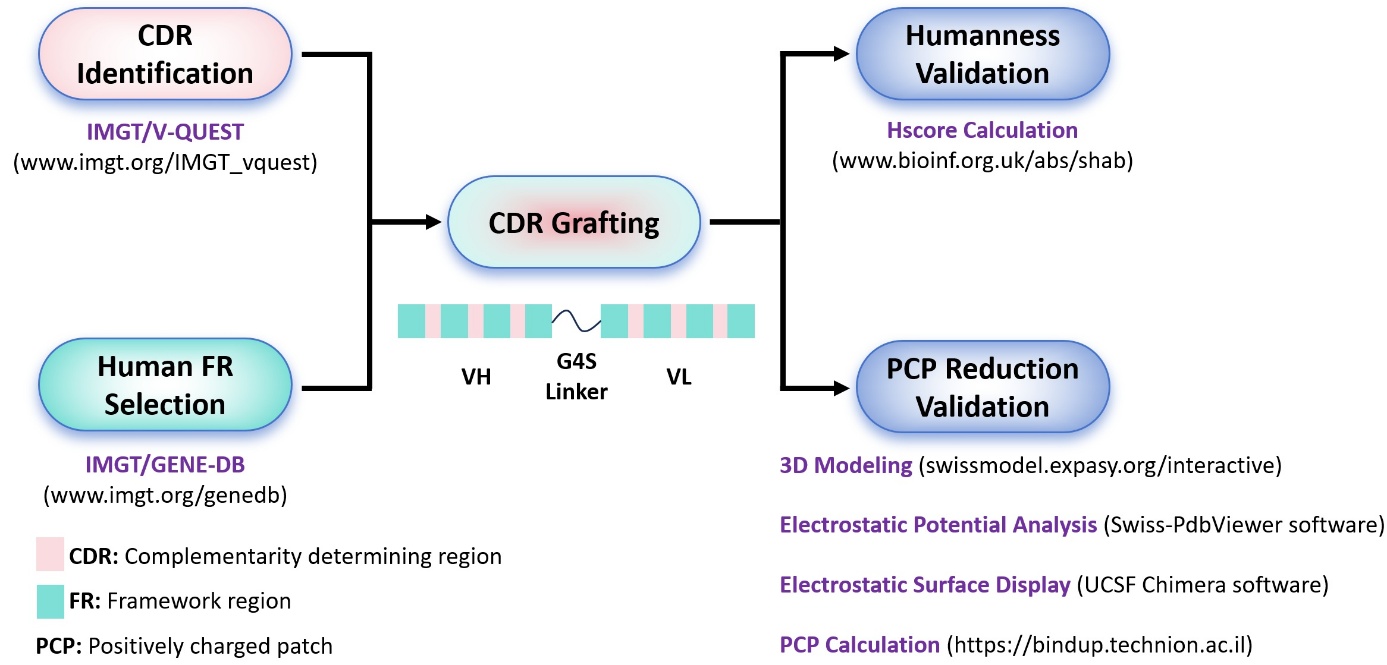


**Figure S10. Computational pipeline for the rational humanization and charge optimization of the CSPG4-specific scFv.**

Murine CDRs identified via IMGT/V-QUEST were grafted onto human germline frameworks selected from IMGT/GENE-DB for low positive-charge density. Sequence humanness was validated using H-score calculations, followed by 3D homology modeling via SWISS-MODEL. Final surface electrostatic characteristics and positively charged patch (PCP) scores were quantified using Swiss-PdbViewer, UCSF Chimera, and BindUp to ensure successful charge reduction.

**Table S1. Regents used in this study.**

| **Reagents** | **SOURCE** | **IDENTIFIER** |
| --- | --- | --- |
| Anti-human CSPG4 (for IHC) | Atlas Antibodies | Cat# HPA002951 |
| Anti-human CSPG4 (for WB) | Abcam | Cat# ab139406; RRID: |
| Anti-human CSPG4 (for FACS) | Santa Cruz | Cat# sc-53389; RRID: AB_784821 |
| Anti-P16-INK4A (F-12) | Santa Cruz | Cat# sc-166760; RRID: AB_2060065 |
| Anti-human CD44 (IM7) | Miltenyi | Cat# 130-095-177; RRID: AB_10839563 |
| Anti-Phospho-Tyrosine (4G10) | Cell Signaling Technology | Cat# 96215 |
| Anti-human β-actin (2D4H5) | Proteintech | Cat# 66009-1-Ig; RRID: AB_2687938 |
| Anti-human IgG-Alexa Fluor 647 | Jackson | Cat#109-605-098; RRID: AB_2337889 |
| Anti-human CD4-BV605 (RPA-T4) | BioLegend | Cat# 300556; RRID: AB_2564391 |
| Anti-human CD8a-PerCP/Cy5.5 (RPA-T8) | BioLegend | Cat# 301032; RRID: AB_893422 |
| Anti-human CD45RA-PE/Cy7 (HI100) | Biolegend | Cat# 304125; RRID: AB_10709440 |
| Anti-human CD62L-PE (DREG-56) | Biolegend | Cat# 304806; RRID: AB_314466 |
| Anti-human CD69-PE (FN50) | Biolegend | Cat# 310906; RRID: AB_314841 |
| Anti-human ICOS-PE-CY7 (C398.4A) | Biolegend | Cat# 313520; RRID: AB_10643411 |
| Anti-human CD25-PerCP-Cy5.5 (BC96) | Biolegend | Cat# 302625; RRID: AB_2125479 |
| Anti-human PD-1-APC (NAT105) | Biolegend | Cat# 367405; RRID: AB_2566066 |
| Anti-human PD-1-PE (eBioJ105) | eBioscience | Cat# 12-2799-42; RRID: AB_11042478 |
| Anti-human LAG-3-PE/Cy7 (3DS233H) | eBioscience | Cat# 12-2239-42; RRID: AB_2572597 |
| Anti-human Tim-3-PerCP/Cy5.5 (F38-2E2) | Biolegend | Cat# 345016; RRID: AB_2561934 |
| Anti-human Ki-67 (Ki-67) | Biolegend | Cat# 350502; RRID: AB_10662385 |
| Recombinant human IL-2 | Beijing Four Rings Biopharmaceutical | Cat# 20200714 |
| TranslT-LT1 | Mirus Bio | Cat# 2300 |
| D-luciferin | Sigma | Cat# L6152 |
| Human T-Activator CD3/CD28 Dynabeads | Thermo Fisher Scientiﬁc | Cat# 11131D |
| Paraformaldehyde | Meilunbio | Cat# MA0192 |
| PrimerScript RT Master Mix | TaKaRa | Cat# 10236505 |
| TB Green Premix Ex Taq | TaKaRa | Cat# 10236504 |
| DAPI | Beyotime | Cat# C1341 |
| Propidium Iodide | Beyotime | Cat# C1008 |
| DMEM | Gibco | Cat# C11995500BT |
| RPMI1640 | Gibco | Cat# 11875093 |
| X-vivo 15 | Lonza | Cat# BE02-054Q |
| Fetal Bovine Serum | Gibco | Cat# A5669701 |
| Trizol | Invitrogen | Cat# 15596018 |
| Penicillin-Streptomycin solution | Gibco | Cat# 15140122 |
| Growth factor-reduced Matrigel | Corning | Cat#354230 |
| Cell Counting Kit-8 | Sigma | Cat#96992 |
| Human T Cell Enrichment Kit | Stem Cell Technology | Cat# 19051 |
| Human IL-2 Uncoated ELISA Kit | Invitrogen | Cat# 88-7025-88; RRID: AB_2574954 |
| Human TNF alpha Uncoated ELISA Kit | Invitrogen | Cat# 88-7346-88; RRID: AB_2575097 |
| Human IFN gamma Uncoated ELISA Kit | Invitrogen | Cat# 88-7316-88; RRID: AB_2575075 |
| Gibson Assembly Master Mix | New England Biolabs | Cat# E2611 |
| Zombie Violet Fixable viability Kit | Biolegend | Cat# 423114 |
| Hematoxylin and Eosin Staining Kit | Beyotime | Cat# C0105 |

**Table S2. Primers used in this study.**

| Primers | SOURCE | IDENTIFIER |
| --- | --- | --- |
| CSPG4 sgRNA-1; F: 5'- CACCGTGGAGCTGTGACACGGAAA; R: 5'- AAACTTTCCGTGTCACAGCTCCAC | This paper | N/A |
| CSPG4 sgRNA-2; F: 5'- CACCGCACCACGCTTACGACACCG; R: 5'- AAACCGGTGTCGTAAGCGTGGTGC | This paper | N/A |
| CSPG4 qPCR; F: 5'- CTGGAGAATGGTGGAAGAG; R: 5'- GGACAGTGACAGTGAAGG | This paper | N/A |
| CD44 shRNA ; 5'-GGACCAATTACCATAACTATT -3; | This paper | N/A |
| GAPDH qPCR; F: 5'- CAAGGTCATCCATGACAACTTTG; R: 5'- GTCCACCACCCTGTTGCTGTAG | This paper | N/A |
| HPV PCR; F: 5'-TTTGTTACTGTGGTAGATACTCAC; R: 5'-GAAAAATAAACTGTAAATCATATTC | Jarmo, Ritari | https://doi.org/10.1371/journal.pone.0034211 |
| Sox2 qPCR; F: 5'-GCACAACTCGGAGATCAGCAAGC; R: 5'-CGGGCAGCGTGTACTTATCCTTC | This paper | N/A |
| Cd34 qPCR; F: 5'-AGGAGAAAGGCTGGGCGAAG; R: 5'-GTTGTCTTGCTGAATGGCCG | This paper | N/A |
| Cd133/Prom1 qPCR; F: 5'-CAATTCACCAGCAACGAGTCCTTC; R: 5'-CTCTCCAACAATCCATTCCCTGTG | This paper | N/A |
| Hmga2 qPCR; F: 5'-TTCCTCAATCACACTACACATCAC; R: 5'-GCAAGTCAATTAGTCCATCTCCTTA | This paper | N/A |
| Ptprz1 qPCR; F: 5'-TCCACACCTCCAACACCTATC; R: 5'-CTGTCTGCTGTAATACCTAAGTCAA | This paper | N/A |
| Bhlhe22 qPCR; F: 5'-GCTCGCCAAGAACTACATCC; R: 5'-GCTGGAGGAGACGCTGTTA | This paper | N/A |
| Igf2bp2 qPCR; F: 5'-AGAGAAGCCTGTCACCATCC; R: 5'-TTCCAATCAGTCTTCCAACCAA | This paper | N/A |
| Vegfa qPCR; F: 5'-GCTTACTCTCACCTGCTTCTG; R: 5'-GCTGCTTCTTCCAACAATGTG | This paper | N/A |
| Ccnd2 qPCR; F: 5'-GCTGTGCATTTACACCGACA; R: 5'-ATGCTTGCGGATCAGAGACA | This paper | N/A |
| Cdc25c qPCR; F: 5'-AGATGCTGGAGGAAGATTCTAAC; R: 5'-TGGATAGCGACAATCAATGACA | This paper | N/A |
| Met qPCR; F: 5'-AGGCACTAGCAAAGTCCGAG; R: 5'-GCCCAGTCTTGTACTCAGCA | This paper | N/A |
| Tex15 qPCR; F: 5'-TTTCACCAGACCATGGGACG; R: 5'-ACGCATCAGGCACAAGAGAA | This paper | N/A |
| Hells qPCR; F: 5'-GAAGAGAAGCCAGTTATGAGGAA; R: 5'-ACACAGAGATTAGTAGAGGAGGAG | This paper | N/A |
| Cxcl2 qPCR; F: 5'-ATCAATGTGACGGCAGGGAAA; R: 5'-TCGAAACCTCTCTGCTCTAACAC | This paper | N/A |
| Alcam qPCR; F: 5'-ATGAGGCAGACGAGATAAGTGA; R: 5'-GCAGCAAGGAGGAGACCAA | This paper | N/A |
| Cldn7 qPCR; F: 5'-CTGTGGGGGAGACGACAAAG; R: 5'-CATACCAGGAGCAAGCTACCA | This paper | N/A |
| Epha7 qPCR; F: 5'-TCATTGCTGTGGTTGCTGTAG; R: 5'-CTTCATCGCCTTCTTGGTCAG | This paper | N/A |
| Slit2 qPCR; F: 5'-CCTCGGAGCAGCAAGCTAAA; R: 5'-TCAGGATCGCCAGCACTAAC | This paper | N/A |
| Aspa qPCR; F: 5'-TCTGTACTTTGCCCTTTGGGTA; R: 5'-TTCTCTGAATCTCAGCGCCA | This paper | N/A |
| Far2 qPCR; F: 5'-GACCTAAGTCAACATTAGTCTACCA; R: 5'-GAGCCGCAGATAGCAGTCA | This paper | N/A |
| Glul qPCR; F: 5'-AGGCATCAAGCAGGTGTA; R: 5'-ACTCAGGCAACTCTTCCA | This paper | N/A |
| Mgll qPCR; F: 5'-CTATCAGACACTGGACCTACCTTA; R: 5'-ACAGCAACCACCTCATCACA | This paper | N/A |
| Pthlh qPCR; F: 5'-CTGCCTGGTTAGACTCTG; R: 5'-AGCCTGTTACCGTGAATC | This paper | N/A |
| Nfib qPCR; F: 5'-CTCAGGCACATCTCAAGCCA; R: 5'-ATTGGCCGGTAAGATGGGTG | This paper | N/A |
| Cbx6 qPCR; F: 5'-GTGCATTTCTCTGTCAAGCCG; R: 5'-ATGTCCTTCTTGAGCCGGTG | This paper | N/A |
| Ceacam1 qPCR; F: 5'-AGAACCAAAGCGACCCCATC; R: 5'-GTCATTGGAGTGGTCCTGCC | This paper | N/A |
| FAK/PTK2 qPCR; F: 5'-TTGGGCGGAAAGAAATCCTG; R: 5'-TGGGGCTGGCTGGATTTTAC | This paper | N/A |
| CD44 qPCR; F: 5'-CTACAAGCACAATCCAGGCAACTC; R: 5'-GTCTTCTTTGGGTGTTTGGCGATA | This paper | N/A |
| OCT4/POU5F1 qPCR; F: 5'-GCCCGAAAGAGAAAGCGAACCA; R: 5'-AAGGAGACCCAGCAGCCTCAAA | This paper | N/A |
| Nanog qPCR; F: 5'-AGCCTCCAGCAGATGCAAGAAC; R: 5'-CCTGCGTCACACCATTGCTATTC | This paper | N/A |
| CD24 qPCR; F: 5'-GCTCCTACCCACGCAGATTTATTC; R: 5'-GAGAGTGAGACCACGAAGAGACTG | This paper | N/A |
| EpCAM qPCR; F: 5'-TATGATCCTGACTGCGATGAGAGC; R: 5'-GTGTCCTTGTCTGTTCTTCTGACC | This paper | N/A |
| CD13/ANPEP qPCR; F: 5'-CCCACCTGGAACTTGAAAGACCTC; R: 5'-CTTGCTGTAGGAGATGGCGTCAA | This paper | N/A |
| Bmi1 qPCR; F: 5'-GTTCACAAGACCAGACCACTAC; R: 5'-GGCAGCATCAGCAGAAGGA | This paper | N/A |
| ALDH1A1 qPCR; F: 5'-AGGATGTTGACAAGGCAGTGAAGG; R: 5'-ATTGACTCCATTGTCGCCAGCAG | This paper | N/A |
| ABCG2 qPCR; F: 5'-TTACAGTTCTCAGCAGCTCTTCGG; R: 5'-CCTCCAGACACACCACGGATAAAC | This paper | N/A |
| ABCB5 qPCR; F: 5'-TATACTGGCATCACTGGTCAATGG; R: 5'-GGCAGCAACACCTATTCCAACATA | This paper | N/A |
| Lin28 qPCR; F: 5'-TTGAGGAGCAGGCAGAGTG; R: 5'-TTGGACAGAGCATGGTTGGA | This paper | N/A |
| satb1 qPCR; F: 5'-GGGGATGTTTCTGCCTTGCT; R: 5'-TGGACCCTTCGGATCACTCA | This paper | N/A |
